# Synthesis and Characterizations of 5,5′‐Bibenzo[*rst*]pentaphene with Axial Chirality and Symmetry‐Breaking Charge Transfer

**DOI:** 10.1002/advs.202200004

**Published:** 2022-02-13

**Authors:** Xiushang Xu, Suman Gunasekaran, Scott Renken, Lorenzo Ripani, Dieter Schollmeyer, Woojae Kim, Massimo Marcaccio, Andrew Musser, Akimitsu Narita

**Affiliations:** ^1^ Max Planck Institute for Polymer Research Ackermannweg 10 Mainz 55128 Germany; ^2^ Organic and Carbon Nanomaterials Unit Okinawa Institute of Science and Technology Graduate University 1919‐1 Tancha, Onna‐son Kunigami‐gun Okinawa 904‐0495 Japan; ^3^ Department of Chemistry & Chemical Biology Cornell University Ithaca NY 14853 USA; ^4^ Dipartimento di Chimica “Giacomo Ciamician” Università di Bologna via Selmi 2 Bologna 40126 Italy; ^5^ Department of Chemistry Johannes Gutenberg University Mainz Duesbergweg 10–14 Mainz 55128 Germany

**Keywords:** axial chirality, benzo[*rst*]pentaphene, biaryl, symmetry‐breaking charge transfer, transient absorption

## Abstract

Exploration of novel biaryls consisting of two polycyclic aromatic hydrocarbon (PAH) units can be an important strategy toward further developments of organic materials with unique properties. In this study, 5,5′‐bibenzo[*rst*]pentaphene (BBPP) with two benzo[*rst*]pentaphene (BPP) units is synthesized in an efficient and versatile approach, and its structure is unambiguously elucidated by X‐ray crystallography. BBPP exhibits axial chirality, and the (*M*)‐ and (*P*)‐enantiomers are resolved by chiral high‐performance liquid chromatography and studied by circular dichroism spectroscopy. These enantiomers have a relatively high isomerization barrier of 43.6 kcal mol^−1^ calculated by density functional theory. The monomer BPP and dimer BBPP are characterized by UV‐vis absorption and fluorescence spectroscopy, cyclic voltammetry, and femtosecond transient absorption spectroscopy. The results indicate that both BPP and BBPP fluoresce from a formally dark S_1_ electronic state that is enabled by Herzberg–Teller intensity borrowing from a neighboring bright S_2_ state. While BPP exhibits a relatively low photoluminescence quantum yield (PLQY), BBPP exhibits a significantly enhanced PLQY due to a greater S_2_ intensity borrowing. Moreover, symmetry‐breaking charge transfer in BBPP is demonstrated by spectroscopic investigations in solvents of different polarity. This suggests high potential for singlet fission in such *π*‐extended biaryls through appropriate molecular design.

## Graphical abstract

5,5′‐Bibenzo[*rst*]pentaphene (BBPP) is synthesized and characterized in comparison to benzo[*rst*]pentaphene (BPP) derivatives. BBPP displays stable axial chirality and can be resolved by chiral high‐performance liquid chromatography. Notably, BBPP demonstrates symmetry‐breaking charge transfer as confirmed by detailed spectroscopic investigations, exhibiting high potential of such *π*‐extended biaryls for singlet fission.

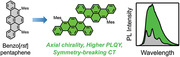

## 1 Introduction

Polycyclic aromatic hydrocarbons (PAHs) have been intensively studied as building blocks to prepare multifunctional organic materials with intriguing properties, exhibiting a great promise for applications in photonics, optoelectronics, and spintronics.^[^ ^1^ ^]^ The electronic structure of PAHs is strongly influenced by symmetry, typically resulting in a complex manifold of bright (optically allowed) and dark (optically forbidden) states. Crucially, the transition probability for these states is governed not by the structural symmetry of the molecule, but rather by the symmetry of the corresponding molecular orbitals. For instance, the transition from the ground (S_0_) to the lowest singlet excited (S_1_) state of tetracene, consisting of four benzene rings fused in a linear fashion (*D*_2h_ structure), is strongly allowed, whereas the transition to the S_2_ state is optically forbidden.^[^ ^2^ ^]^ This and similar molecules typically exhibit mirror‐image symmetry of absorption and emission and negligible Stokes shifts. In contrast, in the case of pyrene, which also consists of four benzene rings but fused in a rhombus‐shape, the situation becomes totally reversed despite the same *D*_2h_ symmetry: the S_0_ to S_1_ transition is forbidden, while the S_0_ to S_2_ transition is allowed.^[^ ^3^ ^]^ In such a case, one might expect the molecule to be essentially nonemissive, as is the case for many linear^[^ ^4^ ^]^ polyenes. However, these optical selection rules can be modulated by coupling to vibrational modes that break the state symmetry. Such Herzberg–Teller vibronic coupling permits dark states to borrow a small but finite oscillator strength from energetically proximate bright states.^[^ ^5^ ^]^ The magnitude of borrowing is determined by the energy difference between bright and dark states. In pyrene, S_1_ and S_2_ are close enough that S_1_ can be directly detected in absorption and efficiently emits photons.^[^ ^6^ ^]^ Such a situation yields complex absorption and emission line shapes, which often cannot be described with simple Franck–Condon progressions.^[^ ^7^ ^]^ Importantly, because of Kasha's rule the principal absorbing and emitting states are different in these systems, and they consequently exhibit deviations from mirror‐image symmetry and much larger Stokes shifts.

**Figure 1:**
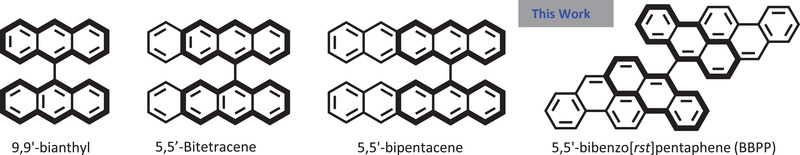
a) Molecular structures of 9,9′‐bianthyl and representative biaryls based on the *π*‐extension of 9,9′‐bianthyl.

The key determining properties, i.e., structural shape, size, and energetic proximity of bright and dark states, are all amenable to tuning through synthetic approaches, making PAHs an excellent platform to modify electronic properties for a variety of applications. Dimerization to form biaryls is a particularly powerful technique to alter their photophysical properties. Depending on the nature of the linkage, the degree of coupling between aryl units can be widely tuned. At the upper limit, this can have the effect of altering the molecular orbital symmetries and thus optical selection rules.^[^ ^8^ ^]^ For intermediate and weaker coupling, interaryl interactions can lead to enhanced delocalization and consequent energetic shifts of the bright states. Moreover, the presence of multiple units opens access to entirely new photophysical decay pathways. For instance, derivatives of 5,5′‐bitetracene and the equivalent 5,5′‐bipentacene (see **Figure** **1**a) exhibit ultrafast singlet fission, in which an initially delocalized singlet state symmetrically separates into two low‐energy triplets, one on each aryl unit.^[^ ^9^ ^]^ In other systems, symmetry‐breaking charge transfer (SB‐CT), a photoinduced electron transfer reaction between two identical chromophores, becomes possible. This phenomenon is of wide interest, since it is a primary process of a bacteriochlorophyll homodimer within the reaction center of natural light‐harvesting systems essential to drive further photochemistry. Similar effects are observed in 9,9′‐bianthryl,^[^ ^10^ ^]^ as well as the aforementioned tetracene and pentacene analogs,^[^ ^9^, ^11^ ^]^ and a host of other dimers.^[^ ^12^ ^]^ SB‐CT can be inferred from solvent‐polarity effects on the fluorescence line‐shape and lifetime,^[^ ^10^, ^11^ ^]^ or directly tracked through the photoinduced absorption of cationic and anionic residues.^[^ ^10a^ ^]^ Though in many instances the photoinduced electron transfer is energetically feasible even in vacuum (Δ*G* = *e*(*E*_ox_ − *E*_red_) − *E*_S1_ < 0), it is typically found that solvent–solute interactions or structural fluctuations are essential to induce the symmetry breaking.^[^ ^9^, ^11^, ^13^ ^]^

Moreover, biaryls can offer inherent chiral properties, as represented by chiral binaphthyls, with various applications ranging from enantioselective catalysis^[^ ^14^ ^]^ and chiroptical devices^[^ ^15^ ^]^ to molecular recognition and switches.^[^ ^16^ ^]^ To obtain stable enantiomers at a room temperature, typical biaryls, such as biphenyls and binaphthyls need bulky substituents to restrict rotation about the axial C—C bond.^[^ ^17^ ^]^ In contrast, functionalized 9,9′‐bianthyls display axial chirality when substituents are introduced at appropriate positions, such as 2,2′‐ and 3,3′‐positions, with high energy barriers (>180 kJ mol^−1^)^[^ ^18^ ^]^ for the isomerization via the rotation at the C9—C9′ bond (Figure 1).^[^ ^18^, ^19^ ^]^ A few other chiral biaryls are reported based on the *π*‐extension of 9,9′‐bianthyl. For examples, 5,5′‐bitetracene exhibits axially chiral properties along C5—C5′ bond and its enantiomers can be resolved by chiral high‐performance liquid chromatography (HPLC; Figure 1).^[^ ^20^ ^]^ Heteroatom‐incorporated analogs, such as bibenzo[*b*]carbazole with anisotropy factors *g*_abs_ on the order of 10^−4^ to 10^−3[^ ^21^ ^]^ and bis(dibenzothiophene)^[^ ^22^ ^]^ are also reported. Nevertheless, reported examples of such axially chiral biaryls based on extended PAHs, without the use of bulky substituents to prohibit the isomerization, are still limited, especially for PAHs with more than four benzene rings.^[^ ^23^ ^]^

Benzo[*rst*]pentaphene (BPP, **1**) is a PAH with a shape of an isosceles triangle, having the two zigzag‐edged sides of equal length and one armchair‐edged side. The synthesis of BPP was reported by Scholl and Neumann in 1922^[^ ^24^ ^]^ and an improved procedure was later described by Clar in 1939.^[^ ^25^ ^]^ BPP has been one of typical PAHs to examine new synthetic methods over the past decades,^[^^]^ with the “dehydrative *π*‐extension (DPEX)” reaction reported by Amsharov and co‐workers in 2018 as one of the latest examples.^[^ ^26^ ^]^ Nevertheless, synthesis of functionalized derivatives of BPP and investigation of their detailed electronic and photophysical properties have rarely been reported.^[^ ^c,27^ ^]^ We have thus selected BPP and its dimer, 5,5′‐bibenzo[*rst*]pentaphene (BBPP),^[^ ^27^ ^]^ as a new platform to explore the SB‐CT effect. BBPP with a structure of bisphenanthro‐fused 9,9′‐bianthryl was also expected to show stable axial chirality.

Herein, we report the synthesis and characterization of 8,8′‐dimesityl‐5,5′‐bibenzo[*rst*]pentaphene (**6**) in comparison to BPP **1**, 5‐mesitylbenzo[*rst*]pentaphene (**3**), and 5,8‐dimesitylbenzo[*rst*]pentaphene (**5**). The structure of **6** was revealed by single‐crystal X‐ray analysis, denoting the presence of enantiomers due to the axial chirality. Density functional theory (DFT) calculations indicated a high isomerization barrier of 43.6 kcal mol^−1^, and the optical resolution could be achieved by chiral HPLC, enabling the analysis by circular dichroism spectroscopy. Through a comprehensive set of spectroscopic and electrochemical measurements, we determine that BPP and its derivatives exhibit similar photophysical properties to pyrene. Namely, all possess a low‐lying dark electronic state which can be weakly detected in absorption due to Herzberg–Teller intensity borrowing. In both monomers and the dimer, this dark S_1_ state is formed by ultrafast (<500 fs) internal conversion and is responsible for the moderately efficient photoluminescence (PL). In BBPP, we detect appreciable interchromophore interactions and a marked increase in fluorescence quantum efficiency. This molecule is uniquely sensitive to solvent polarity, revealing SB‐CT from the dark S_1_ state and subsequent CT emission.

## 2 Results and Discussions

### 2.1 Synthesis

**Scheme 1:**
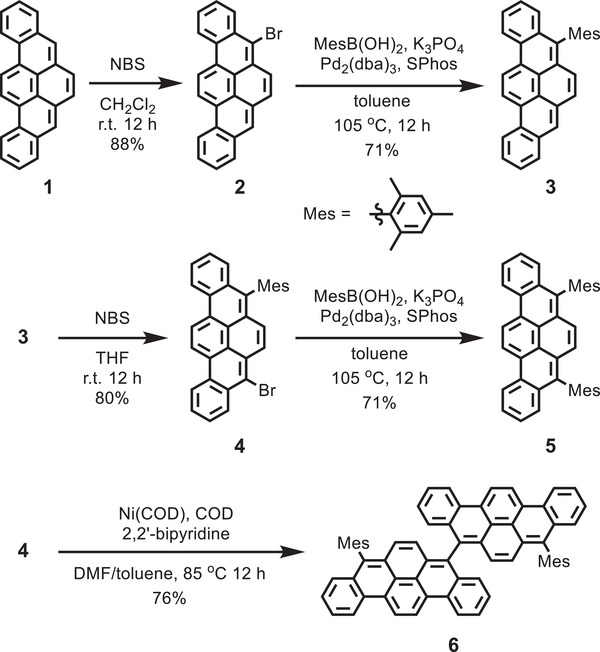
Synthetic route to BPP derivatives **3** and **5**, and 8,8′‐dimesityl‐5,5′‐bibenzo[*rst*]pentaphene (**6**). COD: 1,5‐cyclooctadiene; DMF: dimethylformamide; THF: tetrahydrofuran.

BPP **1** was initially prepared by the “DPEX” reaction according to the literature.^[^ ^26^ ^]^ Subsequently, **1** was brominated by *N*‐bromosuccinimide (NBS) to afford 5‐bromobenzo[*rst*]pentaphene (**2**) in 88% yield (**Scheme** **1**). Next, **2** and mesitylboronic acid were subjected to Suzuki–Miyaura coupling to provide 5‐mesitylbenzo[*rst*]pentaphene (**3**) in 77% yield, where the mesityl (Mes) group was expected to improve solubility and stability. Treatment of **3** with an equivalent amount of NBS then gave 5‐bromo‐8‐mesitylbenzo[*rst*]pentaphene (**4**) in 80% yield, followed by Suzuki–Miyaura coupling with mesitylboronic acid to afford 5,8‐dimesitylbenzo[*rst*]pentaphene (**5**) in 82% yield. On the other hand, **6** was obtained by Yamamoto coupling of **4** in 76% yield. The chemical structures of 8,8′‐dimesityl‐5,5′‐bibenzo[*rst*]pentaphene (**6**) and all the other new compounds were characterized by ^1^H and ^13^C NMR spectroscopy, together with high‐resolution matrix assisted laser desorption ionization‐time of flight mass spectrometry (MALDI‐TOF MS) (see the Supporting Information).

### 2.2 Single‐Crystal X‐Ray Structure of BBPP Dimer

**Figure 2:**
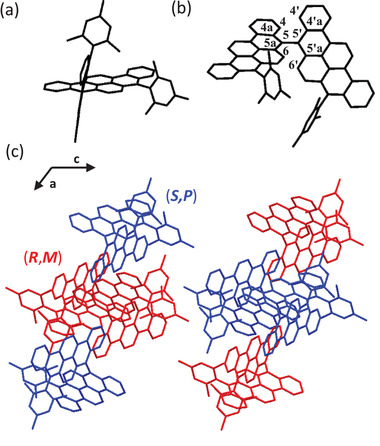
Single‐crystal structure of racemic **6**. a) Side view and b) top view of **6**. c) Molecular packing of **6** in a unit cell. All the hydrogen atoms are omitted for clarity.

The single crystal of racemic **6** was grown by slow evaporation of its solution in CH_2_Cl_2_/CH_3_OH, and X‐ray analysis was carried out at 120 K (**Figure** **2** and Figure S1, Supporting Information). As depicted in Figure 2a, the mesityl group was almost perpendicular to the attached BPP plane, reducing the intermolecular interaction to improve solubility. The two planar BPP moieties were connected to each other through C—C single bond axis with a torsion angle of 84.0^o^ (C5—C4a—C5′—C4a′) (Figure 2b). The steric effect between the hydrogen atoms H4, H6 and H4′, H6′ leads to a nearly orthogonal conformation about the C5—C5′ axis. In a unit cell, enantiomers (*M*)‐ and (*P*)‐enantiomers (Figure S1, Supporting Information) are observed in a ratio of 1:1. As illustrated in Figure 2c, there are two kinds of subunits with four heterochiral molecules being staked alternately along the *c*‐axis. Nevertheless, along the *a*‐axis in every subunit, two homochiral molecules are stacked along the *c*‐axis between another two homochiral molecules. Furthermore, the shortest intermolecular distance between the C—H bonds of the planar BPP and the core of the neighboring BBPP molecules was estimated to be 2.76 Å (Figure S1, Supporting Information). In addition, the face‐to‐face interaction among planar BPPs was not observed, suggesting that **6** was loosely packed in such orthogonal conformations, which reduce the possibility of appearance of excimer. We further confirmed that there is no significant difference between the X‐ray and the DFT‐optimized structures (Figure S2, Supporting Information).

### 2.3 Steady‐State Optical Characterization

**Figure 3:**
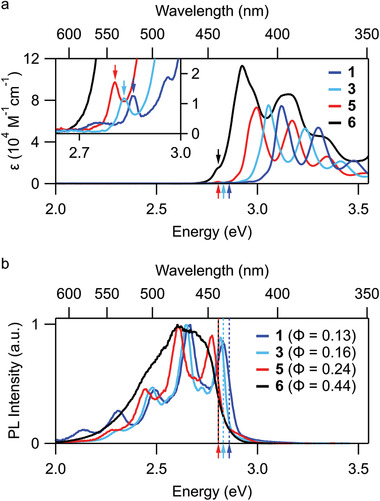
a) Molar absorptivity (*ε*) of compounds **1**, **3**, **5**, and **6** measured in toluene. The forbidden S_1_ absorption peaks of each compound are indicated by arrows, and are highlighted in the inset. b) Normalized PL spectra of compounds **1**, **3**, **5**, and **6** measured in toluene with a 3.5 eV excitation. For reference, the S_1_ absorption energies from (a) are indicated by vertical dashed lines. Since the respective S_1_ states of **5** (red) and **6** (black) coincide in energy, the dashed lines overlap. The absolute PL quantum yields (Φ) are provided in the legend.

The steady‐state absorption spectra for **1**, **3**, **5**, and **6** measured in toluene are presented in **Figure** **3**a. The absorption spectra of the monomeric compounds (**1**, **3**, and **5**) possess three intense absorption peaks (*ε*_max_ ∼ 74 000 M^−1^ cm^−1^) within the measurement window. These three peaks constitute a vibronic progression of a singlet excited state. The absorption peaks reveal redshifts with increasing number of mesityl groups going from **1** to **3** to **5**, due to the electron‐donating property of the mesityl groups with minor delocalization of frontier orbitals (**Table** **1**). Closer inspection of the absorption spectra for **1**, **3**, and **5** reveals that the most intense absorption peaks do not correspond to the lowest energy excited state. Small peaks (*ε* ∼ 1200 M^−1^ cm^−1^) that do not match the primary vibronic progression are observed below the largest absorption peaks for **1**, **3**, and **5** (Figure 3a, inset). These suggest the presence of a lower energy dark singlet state which we denote S_1_. The largest absorption peak and its accompanying vibronic progression can thus be assigned to a distinct bright state, which we denote S_2_. The presence of a dark S_1_ and a bright S_2_ for **1**, **3**, and **5** is reminiscent of pyrene, from which the compounds derive (i.e., benzo[*rst*]pentaphene = dibenzo[*a,i*]pyrene). Drawing analogy from pyrene, the S_0_‐S_1_ transition for **1**, **3**, and **5** is likely symmetry forbidden and absorption is enabled by intensity borrowing from the nearby S_2_ state through Herzberg–Teller vibronic coupling. Time‐dependent DFT (TD‐DFT) calculations at the B3LYP/6‐311G(d,p) level capture well the energetic position of the strongly absorbing state and its systematic redshift from **1** to **3** to **5** (see the Supporting Information). The calculations also reveal a dark state at very similar energies involving the same frontier orbitals, however in each molecule it is predicted to lie slightly above the bright state. We note that computationally reproducing the relative energetic position of these low‐lying states poses a challenge due to their different electronic nature. For instance, TD‐DFT calculations with the B3LYP functional correctly describe the states and ordering in circumpyrene,^[^ ^28^ ^]^ yet fail to correctly place the low‐lying dark state in pyrene.^[^ ^29^ ^]^ Correcting for the contribution of long‐range exchange effects with other functionals results in a correct description of pyrene and an incorrect description of circumpyrene. In this context, we consider the predicted close proximity of bright and dark states to constitute a reasonable description of the molecular electronic structure.

The absorption spectrum of the dimer (**6**) features three primary peaks just as with the monomeric compounds (**1**, **3**, and **5**). Compared to **1**, **3**, and **5**, the three peaks for **6** are further redshifted and are broader. In **6**, the adjacent BPP units (i.e., **3**) behave as coupled chromophores. The redshift in the spectrum of **6** compared to that of **3** is in part due to the long‐range Coulomb interaction between the uncoupled chromophore excitons. The broadening in the peaks can be attributed to excitonic coupling between the chromophores which results in a so‐called Davydov splitting in the transition energies. Indeed, closer inspection of the largest absorption peak for **6** reveals a slight shoulder toward higher energies, suggesting that the absorption peak is a sum of two peaks (Figure 3a). Additionally, the peak molar extinction coefficient of **6** (*ε*_max_ ∼ 113 000 M^−1^ cm^−1^) is less than twice that of **3**, implying a redistribution of the oscillator strength of the individual BPP units due to exciton coupling. The absorption spectrum of **6** also exhibits a small shoulder below the largest absorption peak, indicating a dark S_1_ state similar to the monomeric compounds. Interestingly, in **6** this band exhibits a significantly enhanced molar extinction coefficient (*ε* ∼ 14 000 M^−1^ cm^−1^) compared to the monomers. It occurs at precisely the same spectral position as in monomer **5**, suggesting that excitonic coupling between the two S_1_ states of neighboring monomers is negligible, as we expect for a dark state. This behavior can be explained through the same intensity borrowing mechanism, which depends on the energetic difference between bright and dark states.

**Figure 4:**
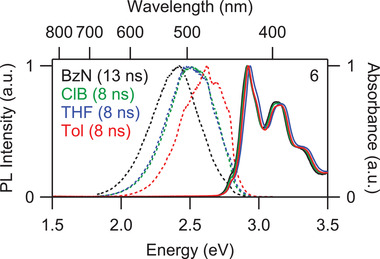
Normalized absorption spectra (solid) and PL spectra (dashed) for **6** in benzonitrile (BzN), chlorobenzene (ClB), tetrahydrofuran (THF), and toluene (Tol).

Additional insights into the photophysical properties of the BPP derivatives can be obtained from the PL spectroscopy. The PL spectra of **1**, **3**, and **5** exhibit structured but more complicated vibronic progressions while the PL spectrum of **6** is unstructured (Figure 3b). The energetic onsets of emission for **1**, **3**, **5**, and **6** correspond well with the energies of the respective forbidden transitions in the absorption spectra (Figure 3a, inset). As noted above, the dark S_1_ state of the BPP monomers acquires a small but nonzero oscillator strength through Herzberg–Teller vibronic coupling. Because S_1_ is also the lowest‐lying singlet state, this effect enables S_1_ to emit photons; the different character of the principal absorbing (S_2_) and emitting (S_1_) states results in a deviation from the standard mirror‐image symmetry typically observed in organic molecules. The absolute PL quantum yields (PLQYs) of monomers **1**, **3**, and **5** are estimated to be 0.13, 0.16, and 0.24, and their PL lifetimes are 17, 20, and 19 ns, respectively (Table 1). Based on these parameters, the radiative rate constants of the monomers are ≈1 × 10^7^ s^−1^ and thus of the same order as in pyrene. Interestingly, the PLQY of the BBPP dimer **6** is substantially larger at 0.44 and the PL lifetime is 8 ns, which is less than half that of the monomers. The lower PL lifetime for **6** can be ascribed to the enhanced radiative rate constant of 5.3 × 10^7^ s^−1^, which is consistent with the increased oscillator strength observed in the absorption spectra. Additionally, the PL spectrum of **6** loses the vibronic structure that is present in the PL spectra of **1**, **3**, and **5** (Figure 3b). This suggests a modified emission mechanism for **6**. With increasing solvent polarity, the absorption spectra of **6** show negligible spectral changes, whereas the PL spectra reveal a considerable redshift (**Figure** **4**). This positive solvatochromism of PL is a hallmark of CT‐state formation upon photoexcitation. Therefore, we assign that **6** undergoes SB‐CT and the degree of stabilization is determined by the solvent polarity.

**Table 1:** Summary of photophysical and energy gap data of all compounds **1**, **3**, **5**, and **6**

	*λ*_abs_ ^a)^ [nm]	*λ*_em_ ^b)^ [nm]	FWHM^b)^ [nm]	*Φ*_PL_ ^c)^	*τ*_PL_ ^d)^ [ns]	*E*_opt_ ^d)^ [eV]	HOMO/LUMO^f)^ [eV]
**1**	375, 397	435, 462	38	0.13	17	2.99	−5.30/−2.06
**3**	383, 405	439, 467	39	0.16	20	2.93	−5.24/−2.05
**5**	390, 414	443, 471	41	0.24	19	2.87	−5.19/−2.04
**6**	394, 423	450	58	0.44	8	2.77	−5.20/−2.09

^a)^ Measured in 10^−5^ m toluene solution. Peak positions correspond to the strongest (S_2_) absorption bands;

^b)^ Measured in 10^−5^ m toluene solution with excitation of their maximum absorption wavelength;

^c)^ Absolute photoluminescence quantum yield measured in toluene solution;

^d)^ PL decay lifetime measured at the emission peak in dilute toluene solution;

^e)^ Optical energy gap (*E*_opt_) was calculated according to the onsets of their UV/vis absorption spectra by using the following equation: *E*_opt_ = 1240/*λ*;

^f)^ HOMO/LUMO energy levels were calculated based on the density functional theory (DFT) at the B3LYP/6‐311G(d,p) level.

### 2.4 Chiral Properties of BBPP Dimer

**Figure 5:**
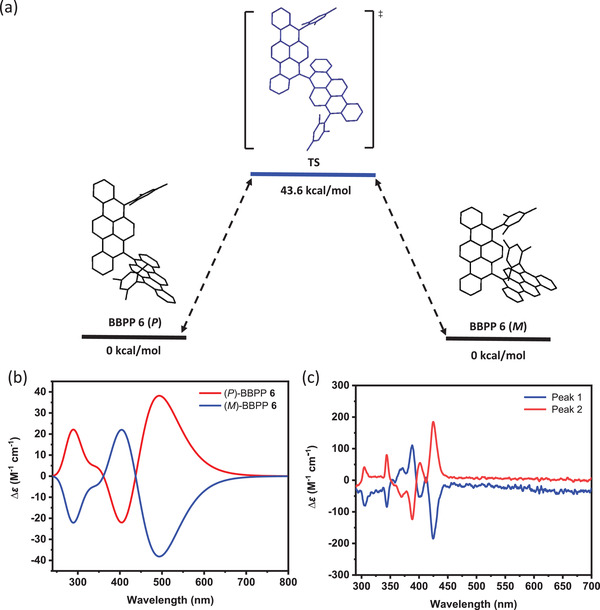
a) *P*/*M* isomerization process of BBPP **6**. The relative Gibbs free energy (kcal mol^−1^) was calculated at the B3LYP/6‐311G(d,p) level. b) Simulated circular dichroism spectra of (*P*)‐isomer to (*M*) by TD‐DFT at the B3LYP/6‐311G(d,p) level. c) Circular dichroism spectra of the first peak and second peak in 10^−6^ m toluene.

The *P*/*M* isomerization process of BBPP **6** was estimated by DFT calculation at the B3LYP/6‐311G‐(d,p) level. As illustrated in **Figure** **5**, the racemization undergoes through a transition state (TS) where the BPP moieties are oriented in an almost parallel manner. The energy barrier for the isomerization via the rotation about C5—C5′ bond is calculated to be 43.6 kcal mol^−1^, encouraging us to separate the enantiomers of BBPP **6**. By using chiral HPLC on Daicel Chiralpak IG column with toluene‐hexane (7:13) as eluent, BBPP **6** could be successfully resolved (Figure S3. Supporting Information), enabling the characterization by circular dichroism (CD) spectroscopy. The obtained experimental CD spectra agreed well with the simulation based on the TD‐DFT calculations (B3LYP/6‐311G(d,p)), allowing the assignment of the first and second eluted peaks in the chiral HPLC separation (Figure S3, Supporting Information) to BBPP **6** (*M*) and BBPP **6** (*P*), respectively (Figure 5b,c). The CD spectrum of BBPP **6** (*P*) is the mirror image of that of BBPP **6** (*M*), resulting from the opposite axial chirality. The major negative and positive Cotton effect of BBPP **6** (*M*) is located at 424 nm (∆*ε* = −183 M^−1^ cm^−1^) and 388 nm (∆*ε* = 108 M^−1^ cm^−1^), respectively. The absorption dissymmetry factor (*g*_abs_ = ∆*ε*/*ε*) of BBPP **6** (*M*) at its absorption maximum (424 nm) was estimated to be 5.56 × 10^−3^ together with its UV‐vis spectrum.

### 2.5 Electrochemical Properties

**Figure 6:**
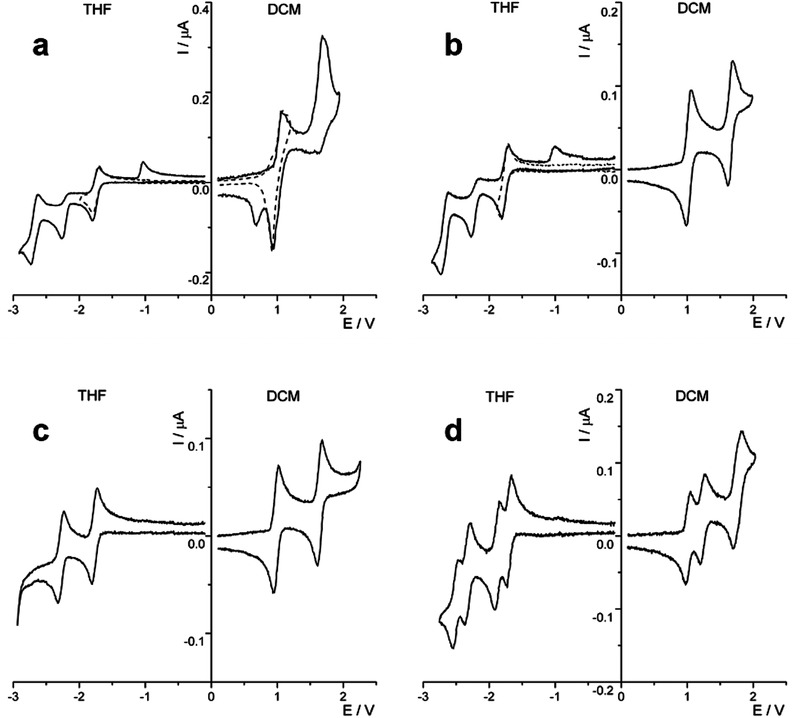
Cyclic voltammetric curves of a) 0.8 × 10^−3^ m compound **1**, b) 0.8 × 10^−3^ m compound **3**, c) 0.7 × 10^−3^ m compound **5**, and d) 0.4 × 10^−3^ m compound **6** recorded in 0.08 m TBAH/DCM and 0.08 m TBAH/THF at 298 K; scan rate: 1 V s^−1^; working electrode: platinum disk (diameter 125 μm); reference electrode: SCE.

The investigation of redox behavior of BPP derivatives **1**, **3**, and **5** and BBPP **6** has been carried out in highly purified and dry dichloromethane (DCM) and tetrahydrofuran (THF) solutions, by exploring potential windows comprised between about +2.0 and −3.0 V. The resulting cyclic voltammetry (CV) curves are reported in **Figure** **6** and the half‐wave (*E* **_½_**) potentials of the various processes, versus saturated calomel electrode (SCE), are summarized in **Table** **2**. Regarding the oxidation processes studied in DCM, BPP **1** shows the first oxidation as a reversible process while the second process is apparently affected by a following up chemical reaction, as indicated by the cathodic peak at 0.70 V, which appears reversing the potential scan soon after the second electron transfer (Figure 6a, full and dashed line curves). However, it must be noticed that, for this compound **1**, both oxidation processes are severely affected by adsorption phenomena, as evidenced by the triangularly shaped peaks and the dependence of peak current on the potential sweep rate. In contrast, BPP **3** shows two one‐electron reversible voltammetric peaks (Figure 6b), indicating that the introduction of a mesityl side‐group on the BPP core makes the compound more stable, without changing the number of voltammetric processes but only modifying slightly their potentials compared to **1**. Similarly, compound **5**, bearing two mesityl groups, shows the same redox pattern as that of **3** (Figure 6c), with potential values shifted to less positive values (about few tens of mV) by the electronic donating effects of mesityl groups (Table 2) in good agreement with the DFT‐calculated highest occupied molecular orbital (HOMO) levels (Table 1). On the other hand, BBPP **6** demonstrated a distinct oxidation pattern comprising three reversible voltammetric peaks (Figure 6d): the first two peaks are one‐electron transfer each, whereas the last voltammetric wave is made of two closely spaced one‐electron transfers. The comparison of the first two oxidations shows an *E* **_½_** separation of 0.22 V, which is a typical value for the interaction between two equivalent redox centers in aromatic species.^[^ ^30^ ^]^ Thus, the first and second oxidation can be ascribed to a one‐electron removal from each BPP moiety of BBPP **6**. The subsequent third and fourth, closely spaced, one‐electron oxidations occur at a potential 0.48 V more positive than the second one, and they represent the abstraction of a second electron from the same molecular orbitals.^[^ ^30^ ^]^ Moreover, these last processes are in the same potential region of the second oxidation for BPP **1**. Interestingly, on the basis of the *E* **_½_** separation, when the trication radical **6** ^3^ **^+•^** is oxidized to tetracation **6** ^4+^, the two BPP moieties have a smaller interaction, i.e., 0.08 V.

**Table 2:** Half‐wave (*E* **_½_**) redox potentials (vs SCE) of compounds **1**, **3**, **5**, and **6** recorded in electrolyte solution at 298 K

Species	Oxid.^a)^ *E*_½_/ V				Red.^b)^ *E*_½_/ V			
	I	II	III	IV	I	II	III	IV
**1**	+1.07^c)^	+1.70^c)^			−1.75	−2.26^c)^	−2.68	
**3**	+1.03	+1.66			−1.76	−2.28^c)^	−2.68	
**5**	+0.98	+1.64			−1.77	−2.28		
**6**	+1.01	+1.23	+1.71^d)^	1.79^d)^	−1.70	−1.88	−2.33	−2.51

^a)^ TBAH/CH_2_Cl_2_ electrolyte solution;

^b)^ TBAH/THF electrolyte solution;

^c)^ Irreversible process; peak potential;

^d)^ *E* **_½_** potential determined by digital simulation of the voltammetric curve.

Concerning the reduction processes investigated in THF, the first one‐electron process is reversible for all the four compounds and occurs at about −1.7 V (Figure 6). The second reduction for BPPs **1** and **3** is complicated by a following up chemical reaction and, as consequence, a small anodic voltammetric peak appears on the reverse scan. At more negative potentials, beyond −2.5 V, a third process is observed, which is reversible although apparently affected by the irreversibility of the second reduction (not further investigated herein). Nevertheless, BPP **5** with two mesityl groups displays a simpler voltammetric pattern with two completely reversible one‐electron reductions (Figure 6c), indicating enhanced chemical stability of the reduced species. Finally, BBPP **6** shows a voltammetric curve with four reversible electron transfers grouped in two set of processes (Figure 6d). Similar to its oxidations, the first two reduction processes are relatively close to each other, with an *E* **_½_** separation of 0.18 V, representing a one‐electron transfer to each BPP moiety. The third reduction is situated at a potential 0.45 V more negative than the second one; it represents the further reduction centered onto one of the two equivalent BPP moieties and hence presumably the electron coupling into one of the two equivalent molecular orbitals. Accordingly, the last reduction occurs at −2.51 V, that is 0.18 V more negative than the third reduction, like the *E* **_½_** separation between the first and second reductions (vide supra).

To estimate the driving force of charge transfer of the dimer **6**,^[^ ^31^ ^]^ we have applied the Weller equation based on the parameters obtained from electrochemical results

(1) $$\begin{array}{ccc} \Delta G_{\mathrm{CT}} & = & \;e\left[E_{\mathrm{ox}}\left(D\right)-E_{\mathrm{red}}\left(A\right)\right]-E_{00}-\frac{e^{2}}{4\pi \varepsilon_{0}\varepsilon_{r}R_{\mathrm{DA}}}\\ & & -\frac{e^{2}}{8\pi \varepsilon_{0}}\left(\frac{1}{r_{D}}+\frac{1}{r_{A}}\right)\left(\frac{1}{\varepsilon_{\mathrm{ref}}}-\frac{1}{\varepsilon_{r}}\right) \end{array}$$

*E*_ox(D)_ and *E*_red(A)_ are the first oxidation and reduction potential energies of BBPP **6**, respectively. Here, we assume that all experiments were done in THF and an oxidation potential value in DCM is nearly the same as that in THF, considering their similar dielectric constants. *E*_00_ is the energy of the state where charge transfer occurs. Here, the S_1_ state of BBPP **6** corresponds to this case and the value is 2.80 eV derived from the second derivative of the linear absorption spectrum (Figure S5, Supporting Information). *R*_DA_ is the center‐to‐center distance between the electron donor and acceptor. Here, we used the distance between the centroids of the two BPPs, which is 7.52 Å. *ε*_r_ and *ε*_ref_ are the dielectric constants of the solvents used in spectroscopic and electrochemical experiments, respectively, which is 7.6. *r*_D_ and *r*_A_ are the effective ionic radii of donor and acceptor. Nevertheless, we do not need to consider these parameters here because the final term, the Born ionic solvation energy, in the Weller equation is zero (since we only consider the situation that the same solvents are used in both electrochemical and spectroscopic experiments). Based on these, we could calculate the Δ*G*_CT_ in THF to −0.34 eV, which identifies that the charge transfer process between the two BPP units of BBPP **6** is thermodynamically favorable. Although we did not estimate Δ*G*_CT_ in other solvents, this value is already negative without any treatment of solvation and electrostatic energies. Therefore, we expect that photoinduced charge transfer of **6** would be feasible regardless of the sort of solvents.

### 2.6 Ultrafast Internal Conversion and Charge Transfer

**Figure 7:**
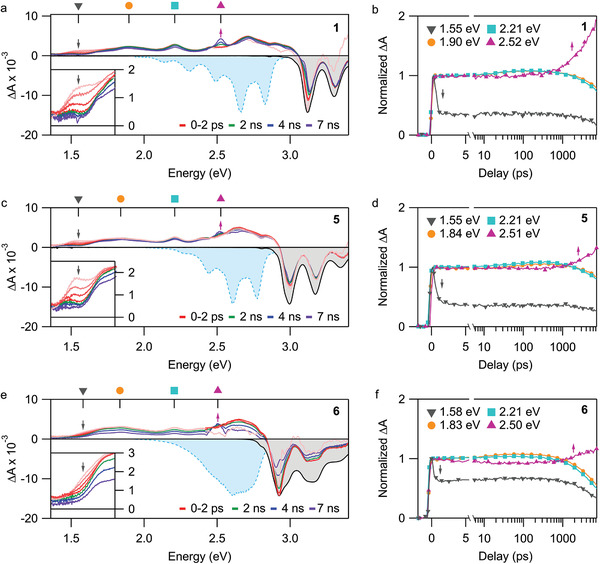
TA spectra in toluene for a) **1** (3.10 eV pump), c) **5** (2.99 eV pump), and e) **6** (2.92 eV pump) as well as kinetic traces at select energies for b) **1**, d) **5**, and f) **6**. In spectral plots, the inverted steady‐state absorption is shaded gray and the inverted steady‐state PL is shaded blue. The probe energies taken for the kinetic traces in (b), (d), and (f) are labeled in the top axis of (a), (c), and (e), respectively.

Transient absorption (TA) spectroscopy was performed on compounds **1**, **5**, and **6** in toluene (≈17 µg mL^−1^). The samples were pumped by a 180 fs pump pulse (150 nJ) at the energy of maximal absorptivity (*ε*_max_). After a time delay (0–7 ns), the samples were probed by an UV probe (2.4–3.4 eV) and a visible (Vis)–near‐infrared (NIR) probe (1.4–2.4 eV). The resulting TA spectra for the two probe regions are combined and presented in **Figure** **7**. Within the UV range, the TA spectra for **1**, **5**, and **6** show clear evidence of ground‐state bleach (GSB), which manifests as a negative Δ*A* signal that overlaps well with the steady‐state absorption. Notably, even though the compounds were pumped at the energy of *ε*_max_, a GSB feature for the S_1_ peak (Figure 3) is observable as a slight modulation at lower energies. This provides confirmation that the S_1_ peak and the S_2_ peaks belong to the same electronic system. Within the Vis–NIR range, there is a broad positive ΔA signal that is due to photoinduced absorption (PIA). There is no discernable evidence of negative stimulated emission (SE) features that match the PL spectra, though the long‐time decay dynamics (right panels) agree well with the measured PL dynamics. We therefore assign these primary TA signatures to the emitting S_1_ state; the absence of SE is consistent with its very weakly emitting nature.

**Figure 8:**
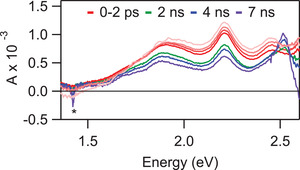
TA spectra of **1** after pumping at 2.86 eV (700 nJ), the energy of the S_1_ peak (Figure 3a). There is no evidence of the initial, rapidly decreasing Δ*A* signal at 1.55 eV observed following higher energy excitation (Figure 7a, inset). The (*) indicates the second‐order diffraction of pump scatter at half the energy of the pump pulse.

At early timescales (0–2 ps), we observe a distinct PIA decay in the 1.45–1.65 eV region for **1** (*τ* = 0.488 ps), **5** (*τ* = 0.530 ps), and **6** (*τ* = 0.234 ps). While these timescales are comparable to vibrational relaxation dynamics, the NIR band does not dynamically shift but rather decays uniformly. This behavior is consistent with ultrafast internal conversion between two electronic states, as widely observed following excitation of the S_2_ state in polyenes.^[^ ^4^, ^33^ ^]^ To probe the origin of this behavior, we further measured the transient absorption of **1** following resonant excitation of the weakly absorbing S_1_ state (**Figure** **8**). We detect the same principal features as observed following higher‐energy S_2_ excitation, except that the fast‐decaying PIA band at 1.55 eV is wholly absent. It is evidently possible to circumvent the ultrafast internal conversion through direct excitation of S_1_, and we accordingly assign the short‐lived band at 1.55 eV as a signature of S_2_. Following the initial sub‐ps evolution, the TA spectra remain relatively constant up to ≈1 ns. Beyond 1 ns, we begin to detect a new PIA peak at ≈2.5 eV. Considering the formation of this band matches the decay of the S_1_ signatures (see right panels in Figure 7), we assign the origin of this peak as the triplet state generated by intersystem crossing.

**Figure 9:**
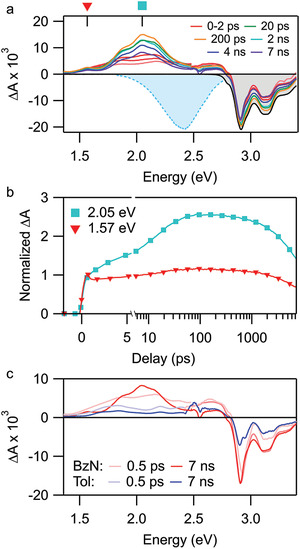
a) TA spectrum of **6** measured in benzonitrile (3.10 eV, 150 nJ). Shaded spectra correspond to inverted steady‐state absorption (gray) and PL (blue). b) Kinetic traces obtained from (a) at two select energies. The energy values correspond to the labels in the top axis of (a). (c) Comparison of the TA spectrum of **6** in toluene versus benzonitrile at short (0.5 ps) and long (7 ns) time delays.

As Figure 7 shows, in toluene dimer **6** follows the same general photophysical pathway as the monomers. By contrast, in benzonitrile we observe an entirely new decay channel for S_1_ (**Figure** **9**). In the 1–100 ps range, we detect the formation of a strong PIA peak centered at 2.05 eV. The formation timescale (12.4 ps) is consistent with solvent reorganization,^[^ ^33^ ^]^ and the decay of this species (≈14 ns) matches the dynamics of the SB‐CT emission reported above. Notably, the TA spectrum of **6** in benzonitrile agrees well with the absorption spectrum of the reduced form (radical anion) of **6**. Together, these results demonstrate rapid, solvent‐induced SB‐CT in **6**, and the absence of any persistent S_1_ features beyond 20 ps indicates this process is essentially quantitative.

## 3 Conclusion

In summary, we have synthesized 8,8′‐dimesityl‐5,5′‐bibenzo[*rst*]pentaphene (BBPP **6**) in a quite facile manner. Compared to the BPP monomers, BBPP **6** exhibits a higher PLQY, a lower HOMO–LUMO (lowest unoccupied molecular orbital) energy gap, and chiral properties. Moreover, the isomerization barrier for BBPP was estimated to be 43.6 kcal mol^−1^, which allows two enantiomers to be resolved by chiral HPLC smoothly. For the monomeric BPPs, we find that the photophysics are governed by a molecular symmetry comparable to pyrene: ultrafast internal conversion into a dark state is followed by vibronically structured emission through Herzberg–Teller coupling. The weak intensity borrowing effect is sufficient to enable direct excitation of the S_1_ state, circumventing the internal conversion pathway. Dimerization to form BBPP results in a significant redshift, pointing to appreciable electronic interactions between the units. However, this perturbation does not alter the optical selection rules and the same basic photophysical progression applies, albeit with markedly higher radiative rate and efficiency due to a proximity‐induced strengthening of the intensity borrowing mechanism. Interestingly, femtosecond transient absorption spectroscopy in polar solution reveals the competing pathway of SB‐CT, which occurs over the timescale of ≈13 ps with nearly quantitative yield. This propensity for charge transfer offers promise for a range of optoelectronic applications. In particular, we highlight that the lowest singlet and triplet state energies calculated by TD‐DFT differ by roughly a factor of 2 in **1**, **5**, and **6**. This condition alone makes them of potential interest for the study of singlet exciton fission, in which a photoexcited singlet exciton can spontaneously separate into two entangled triplet excitons.^[^ ^34^ ^]^ This process can only be accomplished through appreciable coupling between chromophores, of which charge‐transfer‐mediated interactions are among the most widespread. Indeed, SB‐CT as we have observed here is often an essential component for intramolecular singlet fission.^[^ ^9^, ^11^, ^36^ ^]^ We thus suggest that BPP and its dimers may serve as a much‐needed new materials platform for singlet fission studies. We believe that our studying will pave a novel strategy to synthesize and resolve functionalized PAHs via connecting two planar PAHs units.

## 4 Experimental Section

### Synthesis and Basic Characterizations

Details of the synthesis and characterizations of new compounds are reported in the Supporting Information.

### Electrochemical Measurements

For CV measurements, electrochemical or analytical grade tetrabutylammonium hexafluorophosphate (TBAH) from Sigma‐Aldrich was used as received as a supporting electrolyte. Solvent dichloromethane (DCM) from Sigma‐Aldrich and tetrahydrofuran (THF) from Merck were used. DCM was purified and dried by refluxing over and successively distilling from B_2_O_3_ and activated 4 Å molecular sieves.^[^ ^36^ ^]^ THF was treated and purified as previously reported. Both solvents were stored in specially designed Schlenk flasks and protected from light.^[^ ^37^ ^]^ The solvents were distilled via a closed system into a custom designed electrochemical cell (described below) containing the supporting electrolyte and the species under examination, immediately before performing the experiment.

Electrochemical experiments were carried out in an airtight single‐compartment cell using platinum as working and counter electrodes and a silver spiral as a quasi‐reference electrode. The drift of the quasi‐reference electrode was negligible during the time required for an experiment. All the *E*_1⁄2_ potentials were directly obtained from cyclic voltammetric curves as averages of the cathodic and anodic peak potentials and by digital simulation in the case of not Nernstian or overlapping processes. The *E*_1⁄2_ values were determined by adding ferrocene (Fc), at the end of each experiment, as an internal standard and measuring them with respect to the ferrocenium/ferrocene couple (Fc^+/0^) standard potential (at 298 K it was +0.42 V vs SCE, i.e., the aqueous saturated calomel electrode).^[^ ^38^ ^]^

The cell containing the supporting electrolyte and the electroactive compound was dried under vacuum at 100–110 °C for at least 48 h before each experiment. The pressure measured in the electrochemical cell, prior performing the trap‐to‐trap distillation of the solvent, was typically 1 × 10^−5^ mbar. Voltammograms were recorded with a custom made fast and low current potentiostat^[^ ^39^ ^]^ controlled by an AMEL Mod. 568 programmable function generator. The potentiostat was interfaced to a Nicolet Mod. 3091 digital oscilloscope and the data were transferred to a personal computer by the program Antigona.^[^ ^40^ ^]^ Minimization of the uncompensated resistance effect in the voltammetric measurements was achieved by the positive‐feedback circuit of the potentiostat. Digital simulations of the cyclic voltammetric curves were carried out either by Antigona or DigiElch 7, utilizing a best fitting procedure of the experimental curves recorded at different scan rates spanning over, at least, two orders of magnitude.

### Transient Absorption Measurements

Transient absorption measurements were performed with a Helios FIRE spectrometer, driven by a Pharos amplified laser system (Light Conversion) with fundamental 1030 nm operating at 8.5 kHz. The laser output (180 fs) was directed into an automated OPA and harmonic module (Orpheus and Lyra, Light Conversion) to generate narrowband excitation pulses, here in the range 355–465 nm. A portion of the fundamental was separated to generate white light in YAG crystals to cover the UV and Vis–NIR spectral ranges, permitting continuous measurement from 365 to 885 nm. Pump‐probe time delay was set by a mechanical delay stage spanning an 8 ns range.

## Conflict of Interest

The authors declare no conflict of interest.

## Supporting information

Supporting Information

## Data Availability

The data that support the findings of this study are available from the corresponding author upon reasonable request.
