# Supplementary material for: Synthesis and Characterizations of 5,5′‐Bibenzo[rst]pentaphene with Axial Chirality and Symmetry‐Breaking Charge Transfer
Source: Adv Sci (Weinh). 2022 Feb 13;9(19):2200004. doi: 10.1002/advs.202200004 (PMC9259715; doi:10.1002/advs.202200004)
Supplement: Supplementary file 1 — Supporting Information [file ADVS-9-2200004-s001.pdf]

ADVANCED SCIENCE
Open Access

Supporting Information

for *Adv. Sci.*, DOI 10.1002/advs.202200004

Synthesis and Characterizations of 5,5′-Bibenzo[*rst*]pentaphene with Axial Chirality and Symmetry-Breaking Charge Transfer

Xiushang Xu, Suman Gunasekaran, Scott Renken, Lorenzo Ripani, Dieter Schollmeyer, Woojae Kim, Massimo Marcaccio, Andrew Musser and Akimitsu Narita**

ADVANCED SCIENCE
Open Access

Supporting Information

for *Adv. Sci.,* DOI: 10.1002/advs.202200004

Synthesis and Characterizations of 5,5'-Bibenzo[*rst*]pentaphene with Axial Chirality and Symmetry-Breaking Charge Transfer

Xiushang Xu, Suman Gunasekaran, Scott Renken, Lorenzo Ripani, Dieter Schollmeyer, Woojae Kim, Massimo Marcaccio, Andrew Musser, Akimitsu Narita**

Supporting Information for

Synthesis and Characterizations of 5,5'-Bibenzo[*rst*]pentaphene with Axial Chirality and Symmetry-Breaking Charge Transfer

Xiushang Xu,[1,2,+] *Suman Gunasekaran,*[3,+] *Scott Renken,*[3] *Lorenzo Ripani,*[4] *Dieter Schollmeyer,*[5] *Woojae Kim,*[3] *Massimo Marcaccio,*[4] *Andrew Musser,*[*,3] *Akimitsu Narita*[*,1,2]

Dedicated to Professor Dr. Klaus Müllen on the occasion of his 75th birthday.

[1] Max Planck Institute for Polymer Research, 55128 Mainz, Germany.
[2] Organic and Carbon Nanomaterials Unit, Okinawa Institute of Science and Technology Graduate University, 1919-1 Tancha, Onna-son, Kunigami-gun, Okinawa 904-0495, Japan.
[3] Department of Chemistry & Chemical Biology, Cornell University, Ithaca, NY 14850, USA.
[4] Dipartimento di Chimica "Giacomo Ciamician", Università di Bologna, via Selmi 2, 40126 Bologna, Italy.
[5] Department of Chemistry, Johannes Gutenberg University Mainz, Duesbergweg 10–14, 55128 Mainz, Germany.
[+]These authors contributed equally to this work.
E-mail: ajm557@cornell.edu; narita@mpip-mainz.mpg.de; akimitsu.narita@oist.jp

1. General experimental details

All reactions working with air- or moisture-sensitive compounds were carried out under argon atmosphere using standard Schlenk line techniques. All starting materials were purchased from commercial sources and used without further purification. All other reagents were used as received unless otherwise noted. Thin-layer chromatography (TLC) was done on silica gel coated aluminum sheets with F254 indicator and column chromatography separation was performed with silica gel (particle size 0.063-0.200 mm). Nuclear Magnetic Resonance (NMR) spectra were recorded in $C_2D_2Cl_4$, THF-d_8, and $CDCl_3$ using Bruker DPX 300, Bruker DPX 500 MHz NMR spectrometers, respectively. Chemical shifts (δ) were expressed in ppm relative to the residual of solvents ($C_2D_2Cl_4$, ^{1}H: 5.99 ppm, ^{13}C: 74.40 ppm; $CDCl_3$, ^{1}H: 7.26 ppm, ^{13}C: 76.00 ppm; THF-d_8, ^{1}H: 1.72 ppm, 3.52 ppm, ^{13}C: 24.40 ppm, 66.43 ppm). Coupling constants (J) were recorded in Hertz. Abbreviations: s = singlet, d = doublet, t = triplet. High-resolution mass spectra (HR-MS) were recorded on a Bruker Reflex II-TOF spectrometer by matrix-assisted laser decomposition/ionization (MALDI) using 7,7,8,8-tetracyanoquinodimethane (TCNQ) as matrix calibrated with poly(ethylene glycol). UV-vis absorption spectra were recorded on a home-built absorption spectrometer using a Xe plasma as white light source (LDLS, Hamamatsu) and collection on an sCMOS array detector (Kuro, Princeton Instruments). Measurements were performed using 10 mm and 1 mm quartz cells, at room temperature. Steady-state photoluminescence spectra were acquired using an intensified CCD camera (PI-MAX4, Princeton Instruments) coupled to a grating spectrometer (Spectrapro 300, Princeton Instruments) and synchronized to the same Pharos amplifier used for transient absorption, which provided the tunable narrowband excitation pulses 350-450 nm. The same instrument was used for time-resolved photoluminescence, taking advantage of the internal gating function to capture fully spectrally resolved photoluminescence at arbitrary time delays, down to an instrument resolution of 500 ps. For absolute PLQY measurements, we pumped with a CW diode laser at 390 nm. The sample was placed in the center of an integrating sphere and the pump beam was directed on it unfocused. Signal was collected in a fibre coupled into a spectrometer (Ocean Optics Maya Pro 2000). The system response was corrected by measuring a calibrated tungsten lamp and the PLQY was calculated according to the methods described in reference[S1]. Chiral HPLC analysis of 8,8'-dimesityl-5,5'-bibenzo[*rst*]pentaphene eluted by n-hexane/toluene (13:7) using Daicel Chiralpak IG HPLC column. Circular dichroism (CD) spectra were recorded on a JASCO J-820 spectrometer with toluene as a solvent at room temperature.

2. Synthetic Details

Compound **2** was prepared according to our previously reported procedure[S2].

5-mesitylbenzo[*rst*]pentaphene (**3**)

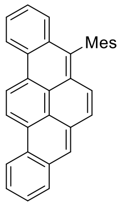

A dried 100-mL Schlenk tube was charged with 5-bromobenzo[*rst*]pentaphene (0.26 g, 0.68 mmol), 2,4,6-trimethylphenylboronic acid (0.17 g, 1.0 mmol), tris(dibenzylideneacetone)dipalladium(0) ($Pd_2(dba)_3$) (32 mg, 34 μmol), 2-dicyclohexylphosphino-2',6'-dimethoxybiphenyl (SPhos) (28 mg, 68 μmol), K_3PO_4 (0.44 g, 2.1 mmol) and anhydrous toluene (20 mL) under argon atmosphere. The reaction mixture was subjected to freeze-pump-thaw cycles (3 times) and heated at 105 ºC for 12 h under argon atmosphere. Then, the resulting mixture was cooled to room temperature and poured into water (50 mL). The organic layer was subsequently separated, and the aqueous layer was extracted with CH_2Cl_2 (2 × 30 mL). The separated organic phases were combined, washed with brine, dried over $MgSO_4$ and evaporated. The residue was purified by silica gel column chromatography (eluent: hexane: CH_2Cl_2 = 10:1) to give the title compound as light yellow solid (0.24 g, 71% yield). ^{1}H NMR (300 MHz, THF-d_8, 298 K) δ 9.33 (q, J = 9.3 Hz, 2H), 9.19 (d, J = 8.4 Hz, 1H), 9.11 (d, J = 8.2 Hz, 1H), 8.32 (s, 1H), 8.22 (d, J = 7.8 Hz, 1H), 7.90 – 7.55 (m, 6H), 7.31 (d, J = 9.5 Hz, 1H), 7.15 (s, 2H), 2.47 (s, 3H), 1.81 (s, 6H). ^{13}C NMR (75 MHz, THF-d_8, 298 K) δ 137.31, 137.21, 134.67, 134.20, 131.63, 130.34, 130.16, 128.71, 128.62, 128.55, 128.29, 128.17, 127.70, 127.57, 126.61, 126.38, 126.25, 126.08, 125.90, 124.70, 123.14, 122.96, 122.24, 121.94, 20.67, 19.50. HR MS (MALDI-TOF): *m*/*z* Calcd. For $C_{33}H_{24}$: 420.1878, $[M]^+$, found: 420.1887.

5-bromo-8-mesitylbenzo[*rst*]pentaphene (**4**)

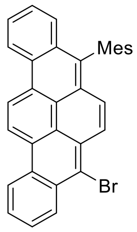

To a solution of 5-mesitylbenzo[*rst*]pentaphene (**3**) (0.17 g, 0.40 mmol) in tetrahydrofuran (THF) (20 mL), a solution of *N*-bromosuccinimide (NBS) (78 mg, 0.44 mmol) in THF (5.0 mL) was added dropwise. The resulting mixture was stirred at room temperature for 12 h. After quenching with acetone (5 mL), the solvents were evaporated and the residue was purified by silica gel column chromatography (eluent: hexane: CH_2Cl_2 = 10:1) to afford tittle compound **4** as yellow solid (0.16 mg, 80% yield). ^{1}H NMR (500 MHz, THF-d_8, 298 K) 9.40 (d, J = 9.2 Hz, 1H), 9.32 (d, J = 9.1 Hz, 1H), 9.18 (m, 2H), 8.80 – 8.72 (m, 1H), 8.25 (d, J = 9.7 Hz, 1H), 7.92 – 7.79 (m, 3H), 7.68 (d, J = 8.2 Hz, 1H), 7.63 (t, J = 7.4 Hz, 1H), 7.46 (d, J = 9.8 Hz, 1H), 7.14 (s, 2H), 2.48 (s, 3H), 1.81 (s, 6H). ^{13}C NMR could not be recorded due to the low solubility of the title compound. HR MS (MALDI-TOF): *m*/*z* Calcd. For $C_{33}H_{23}Br$: 498.0983, $[M]^+$, found: 498.0987.

5,8-dimesitylbenzo[*rst*]pentaphene (**5**)

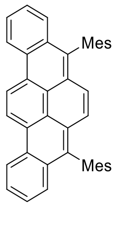

A dried 100-mL Schlenk tube was charged with 5-bromobenzo[*rst*]pentaphene (0.10 g, 0.20 mmol), 2,4,6-trimethylphenylboronic acid (0.066 g, 0.40 mmol), tris(dibenzylideneacetone)dipalladium(0) ($Pd_2(dba)_3$) (9.0 mg, 10 μmol), 2-dicyclohexylphosphino-2',6'-dimethoxybiphenyl (SPhos) (8.2 mg, 20 μmol), K_3PO_4 (0.13 g, 0.60 mmol) and anhydrous toluene (10 mL) under argon atmosphere. The reaction mixture was subjected to freeze-pump-thaw cycles (3 times) and heated

at 105 ºC for 12 h under argon atmosphere. Then, the resulting mixture was cooled to room temperature and poured into water (30 mL). The organic layer was subsequently separated, and the aqueous layer was extracted with CH_2Cl_2 (3 × 20 mL). The separated organic phases were combined, washed with brine, dried over $MgSO_4$ and evaporated. The residue was purified by silica gel column chromatography (eluent: hexane: CH_2Cl_2 = 10:1) to give the title compound as light yellow solid (0.089 g, 82% yield). ^{1}H NMR (300 MHz, $CDCl_3$, 298 K) δ 9.32 (s, 2H), 9.15 (d, J = 8.5 Hz, 2H), 7.81 (dd, J = 7.9, 5.5 Hz, 2H), 7.70 – 7.52 (m, 4H), 7.14 (s, 2H), 7.07 (s, 4H), 2.42 (s, 6H), 1.77 (s, 12H). ^{13}C NMR (75 MHz, $CDCl_3$, 298 K) δ 137.43, 137.22, 134.56, 134.24, 130.37, 128.64, 128.20, 127.72, 127.54, 126.66, 126.42, 126.26, 126.07, 124.82, 123.17, 121.92, 20.98, 19.88. HR MS (MALDI-TOF): *m/z* Calcd. For $C_{42}H_{34}$: 538.2661, [M]$^+$, found: 538.2655.

8,8'-dimesityl-5,5'-bibenzo[*rst*]pentaphene (**6**)

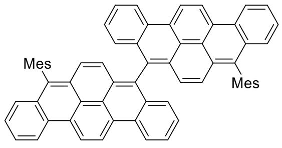

A dried 50-mL Schlenk tube was charged with bis(1,5-cyclooctadiene) nickel(0) (0.15 g, 0.55 mmol), 1,5-cyclooctadiene (60 mg, 0.55 mmol), 2,2′-bipyridine (86 mg, 0.55 mmol) and anhydrous dimethylformamide (DMF) (2.0 mL) under argon atmosphere. The resulting mixture was heated at 85 °C for 30 min under argon atmosphere. Then, this catalyst solution was cooled to room temperature and was added to a solution of 5-bromo-8-mesitylbenzo[*rst*]pentaphene (**4**) (0.28 g, 0.55 mmol) in anhydrous toluene (5 mL) while stirring under argon atmosphere. The resulting mixture was heated at 85 ˚C for 12 h. After cooling to a room temperature, the resulting mixture was poured into water (50 mL). The organic layer was extracted with CH_2Cl_2 (3 × 30 mL), washed with brine, dried over $MgSO_4$, and evaporated. The residue was purified by silica gel column chromatography (eluent: hexane: CH_2Cl_2 = 5:1) to give the title compound as yellow solid (0.18 g, 76% yield). ^{1}H NMR (500 MHz, $C_2D_2Cl_4$, 403 K) δ 9.40 (s, 4H), 9.22 (d, J = 8.5 Hz, 2H), 9.17 (d, J = 8.6 Hz, 2H), 7.83 (t, J = 7.8 Hz, 2H), 7.78 (t, J = 7.7 Hz, 2H), 7.71 (d, J = 8.4 Hz, 2H), 7.62 (t, J = 7.6 Hz, 2H), 7.54 (d, J = 8.4 Hz, 2H), 7.43 (t, J = 7.6 Hz, 2H), 7.01 – 6.87 (m, 8H), 2.33 (s, 6H), 1.76 (s, 12H). ^{13}C NMR (126 MHz, $C_2D_2Cl_4$, 403 K) δ 137.46, 137.12, 135.04, 134.65, 132.29, 131.89, 130.88, 130.21, 129.08, 128.90, 128.65, 128.25, 128.06, 127.88, 127.72, 126.99, 126.77, 126.73, 126.62, 126.11, 126.07, 125.08, 123.19, 122.49, 122.05, 20.89, 19.85. HR MS (MALDI-TOF): *m/z* Calcd. For $C_{66}H_{46}$: 838.3600 [M]$^+$, found: 838.3611.

3. X-ray single crystallography

The single crystal of compound **6** suitable for X-ray analysis was obtained by slow evaporation of its solution in CH_2Cl_2/CH_3OH. The structure was deposited at the Cambridge Crystallographic Data Centre (CCDC) and the data could be obtained free of charge via www.ccdc.cam.ac.uk/structures.

Crystal data for compound **6** (CCDC number: 2131090)

formula	$C_{66}H_{46}$ + solvent		
molecular weight	839.07 gmol^{-1}		
absorption	μ = 0.47 mm^{-1}		
transmission	T_{min} = 0.8944, T_{max} = 0.9912		
crystal size	0.02 x 0.07 x 0.44 mm^3 yellow needle		
space group	I 2/a (monoclinic)		
lattice parameters	a =16.053(2)Å		
(calculate from	b =17.2192(15)Å	ß = 95.146(11)°	
12895 reflections with	c =36.734(5)Å		
2.9° < θ < 68.3°)	V = 10113(2)Å^3	z = 8	F(000) = 3536
temperature	120 K		
density	d_{xray} = 1.102 gcm^{-3}		

data collection

diffractometer	STOE IPDS 2T
radiation	Cu-K_α IµS mirror system
Scan – type	ω scans
Scan – width	1°
scan range	2.4° ≤ θ < 68°<br>-18 ≤ h ≤ 18 -18 ≤ k ≤ 17 -41 ≤ l ≤ 42
number of reflections:	
measured	20458
unique	8462 (R_{int} = 0.1276)
observed	3356 (|F|/σ(F) > 4.0)

data correction, structure solution, and refinement

corrections	Lorentz and polarisation correction.
Structure solution	Program: SIR-2004 (Direct methods)
refinement	Program: SHELXL-2018 (full matrix). 602 refined parameters, weighting scheme:<br>$w=1/[\sigma^2(F_o^2) + (0.2*P)^2]$<br>with $(Max(F_o^2,0)+2*F_c^2)/3$. H-atoms at calculated positions and refined with isotropic displacement parameters, non H- atoms refined anisotropically.
R-values	wR2 = 0.6084 (R1 = 0.23 for observed reflections, 0.3483 for all reflections)
goodness of fit	S = 1.748
maximum deviation of parameters	0.001 * e.s.d
maximum peak height in diff. Fourier synthesis	0.85, -0.59 eÅ^{-3}
remark	structure contains unknown amount of solvent

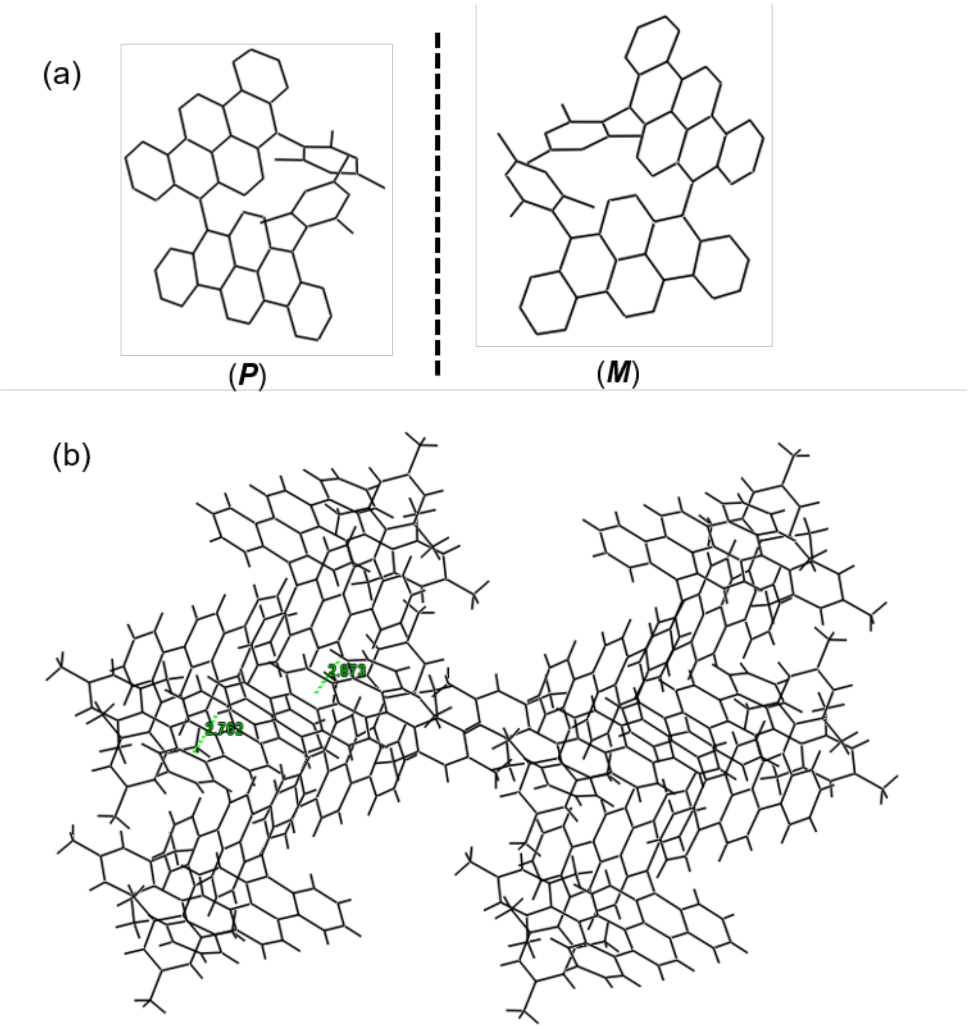

Figure S1. Single-crystal structure of racemic **6**. (a) Absolute configurations of (*P*) -and (*M*) -isomer; (b) packing arrangement of **6** in the crystal (solvents are omitted for clarity).

4. DFT calculations

DFT calculations were performed using the Gaussian 09 software package[S3]. The geometries, molecular orbitals, and MO energies were calculated at the B3LYP/6-311G(d,p) level. The transition state (TS)-structure of **6** was also calculated at B3LYP/6-311G(d,p) level and its imaginary frequency was estimated to be –11.93 cm^{-1}.

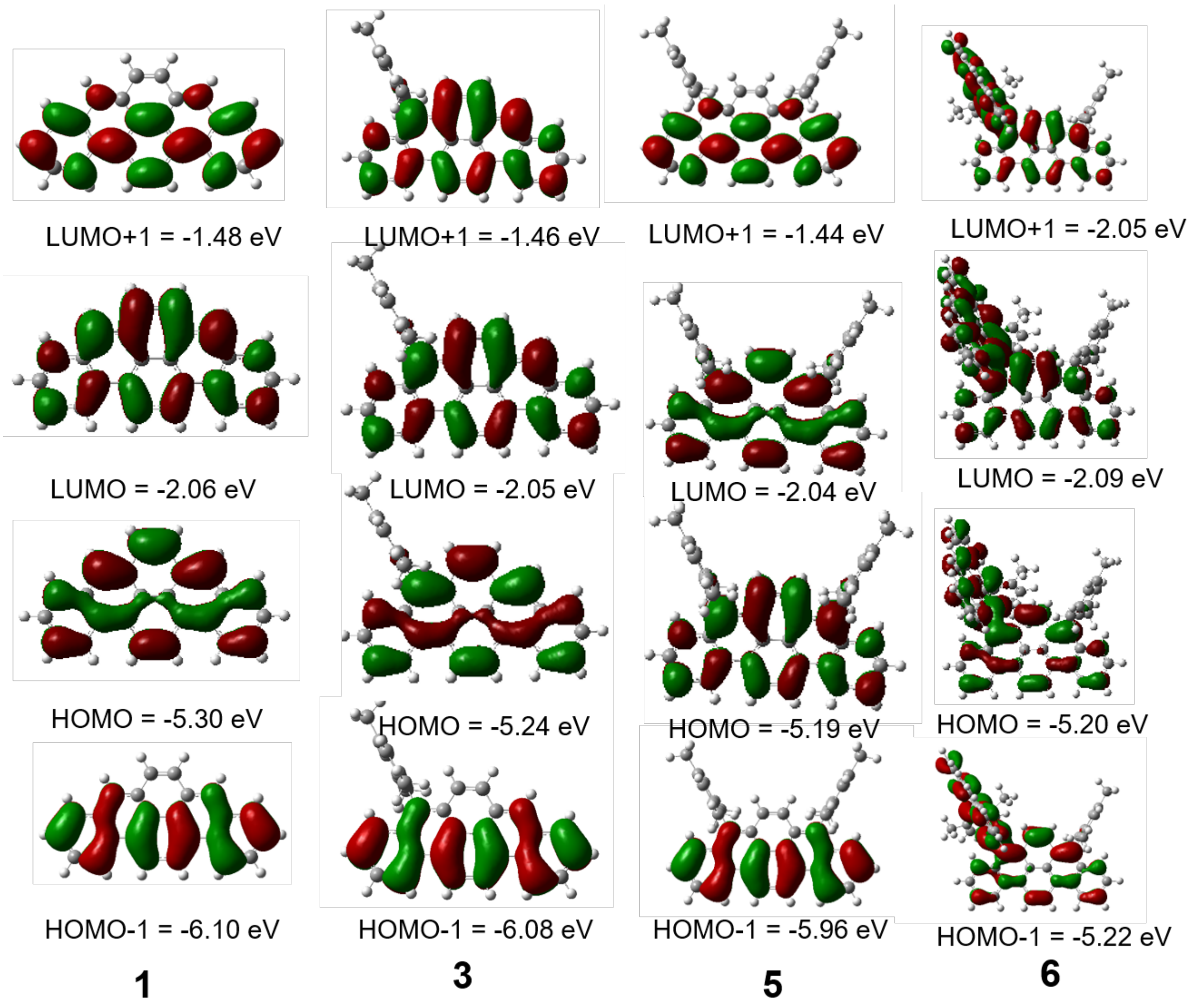

Figure S2. Molecular orbitals and HOMO-1, HOMO, LUMO, and LUMO + 1 energy diagrams of **1, 3, 5,** and **6**.

Table S1. Characteristic electron transitions for **1**, **3**, **5**, and **6** were calculated by TDDFT at the B3LYP/6-311G(d,p) level by Gaussian 09.

Compound	Excited states	Transition energy (eV)	Wavelength (nm)	Oscillator strength	Description
**1**	S1	3.0870	402	0.4920	HOMO-1 → LUMO+1 -0.16965<br>HOMO → LUMO 0.67959
	S2	3.1358	396	0.0029	HOMO-1 → LUMO 0.39691<br>HOMO → LUMO+1 0.67959
	S3	3.6723	338	0.0784	HOMO-1 → LUMO 0.57159<br>HOMO → LUMO+1 -0.39336
**3**	S1	3.0288	409	0.5634	HOMO-1 → LUMO+1 0.15832<br>HOMO → LUMO 0.68264
	S2	3.1085	399	0.0036	HOMO-1 → LUMO -0.39423<br>HOMO → LUMO+1 0.58005
	S3	3.6307	341	0.1005	HOMO-1 → LUMO 0.57400<br>HOMO → LUMO+1 0.39038
**5**	S1	2.9690	417	0.6282	HOMO-1 → LUMO+1 -0.14783<br>HOMO → LUMO 0.68570
	S2	3.0819	402	0.0043	HOMO-1 → LUMO 0.39227<br>HOMO → LUMO+1 0.58144
	S3	3.5888	345	0.1262	HOMO-1 → LUMO 0.57630<br>HOMO → LUMO+1 -0.38868
**6**	S1	2.6675	465	0.0054	HOMO-1 → LUMO+1 -0.45075<br>HOMO → LUMO 0.53797
	S2	2.6686	465	0.0012	HOMO-1 → LUMO 0.50946<br>HOMO → LUMO+1 -0.48277
	S3	2.8804	430	0.9370	HOMO-1 → LUMO 0.53017<br>HOMO → LUMO+1 0.44279

Table S2. Cartesian coordinates of the DFT-optimized **1**.

Tag	Symbol	X	Y	Z
1	C	2.8052	1.726984	-0.000003
2	C	-2.8052	1.726984	-0.000001
3	C	-3.670587	-1.906995	0.000006
4	C	-5.046937	-1.849426	0.000012
5	C	-5.709937	-0.605584	0.00001
6	C	-4.976629	0.556101	0.000005
7	C	-3.55832	0.526711	0
8	C	3.55832	0.526711	-0.000001
9	C	4.976629	0.556101	0.000003
10	C	5.709937	-0.605584	0.00001
11	C	5.046937	-1.849426	0.000014
12	C	3.670587	-1.906995	0.000009
13	C	-2.881518	-0.731351	-0.000001
14	C	2.881518	-0.731351	-0.000001
15	C	1.434691	-0.747745	-0.000008
16	C	0.683765	-1.953643	-0.000021
17	C	-0.683766	-1.953643	-0.000021
18	C	-1.434691	-0.747744	-0.000008
19	C	1.42796	1.726877	-0.000002
20	C	0.714779	0.472221	-0.000004
21	C	-0.714779	0.472221	-0.000004
22	C	-1.42796	1.726877	-0.000001
23	C	-0.674977	2.958331	0.000001
24	C	0.674977	2.958331	0
25	H	3.336951	2.673448	-0.000003

26	H	-3.336951	2.673448	0
27	H	-3.193175	-2.878025	0.00001
28	H	-5.623772	-2.767344	0.000018
29	H	-6.793403	-0.569415	0.000014
30	H	-5.474561	1.520088	0.000005
31	H	5.474561	1.520088	0.000001
32	H	6.793403	-0.569415	0.000013
33	H	5.623772	-2.767344	0.000023
34	H	3.193176	-2.878025	0.000016
35	H	1.197071	-2.905904	-0.000035
36	H	-1.197071	-2.905904	-0.000035
37	H	-1.227075	3.892261	0.000003
38	H	1.227075	3.892261	0.000002

Table S3. Cartesian coordinates of the DFT-optimized **3**.

Tag	Symbol	X	Y	Z
1	C	-1.639663	0.280754	0.000005
2	C	3.378761	-2.283672	-0.000005
3	C	5.810636	0.54941	-0.000002
4	C	7.008238	-0.131332	-0.000002
5	C	7.029062	-1.540755	-0.000004
6	C	5.845623	-2.238239	-0.000005
7	C	4.597935	-1.562976	-0.000005
8	C	-1.730357	1.710916	0.000006
9	C	-2.992642	2.364296	0.00001
10	C	-3.089844	3.735376	0.00001
11	C	-1.921946	4.520925	0.000006
12	C	-0.68476	3.917395	0.000002
13	C	4.570916	-0.134899	-0.000003
14	C	-0.544123	2.508036	0.000003
15	C	0.746	1.857159	0
16	C	1.96651	2.583986	-0.000002
17	C	3.181157	1.957671	-0.000003
18	C	3.291886	0.542241	-0.000003
19	C	-0.3968	-0.34024	0.000001
20	C	0.81737	0.444306	0
21	C	2.091182	-0.210551	-0.000002
22	C	2.155101	-1.651172	-0.000004
23	C	0.922894	-2.397773	-0.000003
24	C	-0.278784	-1.78199	-0.000001
25	C	-2.898581	-0.536231	0.000007
26	C	-3.494025	-0.915496	1.21858
27	C	-4.670904	-1.667437	1.195615
28	C	-5.275196	-2.056309	0.000007
29	C	-4.670915	-1.667411	-1.195611
30	C	-3.494044	-0.915477	-1.218574
31	C	-2.879653	-0.52271	-2.542221
32	C	-2.879625	-0.522757	2.542231
33	C	-6.53023	-2.896052	-0.000021
34	H	3.415853	-3.368648	-0.000006
35	H	5.830921	1.631209	0
36	H	7.940994	0.421205	0
37	H	7.976052	-2.068436	-0.000004
38	H	5.847112	-3.323235	-0.000006
39	H	-3.889697	1.758403	0.000014
40	H	-4.064112	4.211056	0.000013
41	H	-1.995238	5.602594	0.000005

42	H	0.195612	4.545824	-0.000001
43	H	1.948491	3.665268	-0.000001
44	H	4.074332	2.567835	-0.000004
45	H	0.983564	-3.481152	-0.000005
46	H	-1.189512	-2.367076	0
47	H	-5.127021	-1.953996	2.138804
48	H	-5.127044	-1.953952	-2.138801
49	H	-3.492538	-0.87545	-3.373729
50	H	-2.778763	0.562533	-2.631857
51	H	-1.877158	-0.944695	-2.658458
52	H	-2.778717	0.562482	2.631883
53	H	-3.492514	-0.875499	3.373735
54	H	-1.877137	-0.944762	2.65846
55	H	-7.140604	-2.701204	0.884923
56	H	-7.141673	-2.699553	-0.88387
57	H	-6.288273	-3.964693	-0.001182

Table S4. Cartesian coordinates of the DFT-optimized **5**.

Tag	Symbol	X	Y	Z
1	C	-0.67533	1.018205	0.000006
2	C	0.675328	1.018205	0.000008
3	C	1.440986	-0.20591	0.000006
4	C	0.71755	-1.45618	0.000003
5	C	-0.717552	-1.456179	0.000005
6	C	-1.440989	-0.205909	0.000005
7	C	1.430728	-2.678612	-0.000001
8	C	0.682843	-3.885355	0.000002
9	C	-0.682846	-3.885355	0.000006
10	C	-1.43073	-2.678612	0.000006
11	C	-2.876001	-2.666597	0.000008
12	C	2.875999	-2.666597	-0.000007
13	C	-3.646202	-3.855425	0.000011
14	C	-5.022444	-3.826037	0.000012
15	C	-5.701796	-2.593261	0.00001
16	C	-4.988045	-1.418692	0.000006
17	C	-3.566604	-1.415659	0.000005
18	C	3.566601	-1.41566	-0.000003
19	C	4.988043	-1.418692	-0.000007
20	C	5.701793	-2.593261	-0.000017
21	C	5.022441	-3.826037	-0.000024
22	C	3.646199	-3.855425	-0.000018
23	C	2.830483	-0.187154	0.000005
24	C	-2.830486	-0.187154	0.000002
25	C	3.574025	1.11649	0.000011
26	C	3.924554	1.729415	1.218476
27	C	4.618059	2.941635	1.195598
28	C	4.977254	3.564039	0.000019
29	C	4.618062	2.941635	-1.195576
30	C	3.924564	1.729426	-1.218461
31	C	3.554195	1.101201	-2.542055
32	C	3.554173	1.1012	2.542072
33	C	5.755572	4.858077	-0.000009
34	C	-3.574027	1.116491	-0.000004
35	C	-3.924556	1.72943	1.218462
36	C	-4.618054	2.941647	1.195575
37	C	-4.977248	3.564045	-0.000016
38	C	-4.618061	2.941631	-1.195599

39	C	-3.924562	1.729414	-1.218475
40	C	-3.55419	1.101187	-2.542067
41	C	-3.554176	1.101219	2.54206
42	C	-5.755561	4.858085	-0.000019
43	H	-1.214545	1.956971	0.000005
44	H	1.214543	1.95697	0.00001
45	H	1.195322	-4.837529	0.000003
46	H	-1.195325	-4.837529	0.000009
47	H	-3.151485	-4.817266	0.000013
48	H	-5.582367	-4.754418	0.000015
49	H	-6.785766	-2.570657	0.000011
50	H	-5.508006	-0.469228	0.000005
51	H	5.508003	-0.469229	-0.000001
52	H	6.785764	-2.570657	-0.000021
53	H	5.582364	-4.754419	-0.000033
54	H	3.151482	-4.817266	-0.000024
55	H	4.881899	3.411235	2.138806
56	H	4.881904	3.41124	-2.138783
57	H	3.936142	1.69614	-3.373718
58	H	3.960421	0.090011	-2.633783
59	H	2.469366	1.018431	-2.655718
60	H	3.960311	0.089973	2.633771
61	H	3.936198	1.696093	3.373732
62	H	2.469339	1.018526	2.655768
63	H	5.531505	5.458259	0.884992
64	H	6.834672	4.668043	-0.001344
65	H	5.529551	5.459401	-0.883746
66	H	-4.881891	3.411256	2.13878
67	H	-4.881904	3.411228	-2.138808
68	H	-3.936188	1.696091	-3.373731
69	H	-2.469358	1.018479	-2.655753
70	H	-3.960358	0.089973	-2.633769
71	H	-2.469342	1.018543	2.655756
72	H	-3.936198	1.696117	3.373718
73	H	-3.960316	0.089994	2.633764
74	H	-5.530424	5.458893	0.884291
75	H	-5.530603	5.458782	-0.884448
76	H	-6.834662	4.668056	0.000104

Table S5. Cartesian coordinates of the DFT-optimized **6**.

Tag	Symbol	X	Y	Z
1	C	0.556695	2.331503	0.503515
2	C	4.969563	-1.143247	-0.19728
3	C	7.118157	-0.341621	2.779899
4	C	8.178936	-1.197038	2.585359
5	C	8.200091	-2.052041	1.46742
6	C	7.159206	-2.031027	0.570137
7	C	6.04988	-1.159267	0.742946
8	C	0.487861	3.220773	1.62415
9	C	-0.618951	4.094038	1.802473
10	C	-0.690206	4.948627	2.875907
11	C	0.347686	4.968385	3.826562
12	C	1.43256	4.13394	3.681388
13	C	6.027396	-0.29108	1.877595
14	C	1.542585	3.240083	2.587913
15	C	2.674331	2.35707	2.418811
16	C	3.755208	2.326625	3.33891

17	C	4.819836	1.488119	3.170236
18	C	4.905502	0.600336	2.065709
19	C	1.647534	1.484926	0.344829
20	C	2.72672	1.485009	1.305197
21	C	3.845646	0.604096	1.128021
22	C	3.89409	-0.283336	-0.010402
23	C	2.793222	-0.246719	-0.943662
24	C	1.740326	0.582072	-0.777713
25	C	-1.740328	0.582075	0.777715
26	C	-2.793224	-0.246716	0.943664
27	C	-3.894092	-0.283335	0.010403
28	C	-3.845646	0.604094	-1.128022
29	C	-2.72672	1.485006	-1.305199
30	C	-1.647535	1.484926	-0.344829
31	C	-4.905501	0.600332	-2.065711
32	C	-4.819834	1.488112	-3.17024
33	C	-3.755206	2.326618	-3.338915
34	C	-2.67433	2.357065	-2.418815
35	C	-1.542584	3.240078	-2.587918
36	C	-6.027395	-0.291084	-1.877597
37	C	-1.432558	4.133933	-3.681394
38	C	-0.347684	4.968377	-3.826569
39	C	0.690207	4.948621	-2.875913
40	C	0.618952	4.094034	-1.802477
41	C	-0.48786	3.22077	-1.624154
42	C	-6.049881	-1.159269	-0.742946
43	C	-7.159206	-2.031029	-0.570136
44	C	-8.20009	-2.052045	-1.467421
45	C	-8.178934	-1.197045	-2.585362
46	C	-7.118155	-0.341628	-2.779902
47	C	-4.969564	-1.143246	0.197282
48	C	-0.556696	2.331502	-0.503517
49	C	5.011967	-2.062366	-1.382858
50	C	4.465085	-3.356973	-1.292197
51	C	4.524192	-4.199674	-2.40447
52	C	5.108257	-3.796918	-3.605513
53	C	5.64691	-2.511987	-3.672917
54	C	5.609403	-1.636826	-2.584998
55	C	6.204303	-0.253578	-2.713373
56	C	3.817627	-3.842486	-0.015947
57	C	5.131545	-4.714002	-4.804933
58	C	-5.011969	-2.062363	1.382861
59	C	-4.465082	-3.356969	1.292205
60	C	-4.52419	-4.199666	2.404479
61	C	-5.108258	-3.796909	3.605521
62	C	-5.646916	-2.51198	3.67292
63	C	-5.60941	-1.636822	2.584999
64	C	-6.204314	-0.253575	2.71337
65	C	-3.817619	-3.842482	0.015958
66	C	-5.131541	-4.713992	4.804942
67	H	7.129314	0.303701	3.647836
68	H	8.998403	-1.211078	3.295059
69	H	9.035843	-2.725594	1.314738
70	H	7.17301	-2.687487	-0.290491
71	H	-1.417465	4.077505	1.072409
72	H	-1.544479	5.606113	2.990771
73	H	0.29364	5.64105	4.675076
74	H	2.214465	4.170321	4.42775

75	H	3.748332	2.979506	4.200779
76	H	5.612009	1.511675	3.905905
77	H	2.827053	-0.910052	-1.798465
78	H	0.934561	0.579185	-1.500014
79	H	-0.934564	0.57919	1.500016
80	H	-2.827057	-0.910046	1.798469
81	H	-5.612006	1.511667	-3.90591
82	H	-3.74833	2.979497	-4.200785
83	H	-2.214463	4.170312	-4.427757
84	H	-0.293637	5.64104	-4.675085
85	H	1.54448	5.606107	-2.990778
86	H	1.417465	4.077503	-1.072413
87	H	-7.173011	-2.687487	0.290493
88	H	-9.035842	-2.725599	-1.314739
89	H	-8.9984	-1.211087	-3.295063
90	H	-7.129312	0.303692	-3.647841
91	H	4.103014	-5.197879	-2.327373
92	H	6.111512	-2.178822	-4.596433
93	H	6.654433	-0.115429	-3.698136
94	H	5.444687	0.521807	-2.577656
95	H	6.97654	-0.076582	-1.959546
96	H	2.950071	-3.231341	0.249389
97	H	3.482466	-4.87602	-0.120523
98	H	4.509936	-3.794331	0.829262
99	H	5.169167	-5.763609	-4.503917
100	H	4.233115	-4.581938	-5.41801
101	H	5.994611	-4.513125	-5.444015
102	H	-4.10301	-5.197871	2.327386
103	H	-6.111521	-2.178815	4.596434
104	H	-6.654451	-0.115428	3.698129
105	H	-5.444699	0.521811	2.577658
106	H	-6.976546	-0.076582	1.959538
107	H	-2.95005	-3.231348	-0.249363
108	H	-3.482475	-4.876021	0.12053
109	H	-4.509917	-3.794308	-0.829258
110	H	-5.169314	-5.763595	4.503927
111	H	-4.233038	-4.582035	5.417934
112	H	-5.994525	-4.513016	5.444105

Table S6. Cartesian coordinates of the DFT-optimized transition state (TS) structure of **6**.

Tag	Symbol	X	Y	Z
1	C	2.903768	1.28549	0.74888
2	C	4.187495	0.98052	0.441846
3	C	4.613495	-0.37477	0.220884
4	C	3.676794	-1.41665	0.557628
5	C	2.354951	-1.07917	1.00787
6	C	1.880802	0.288793	0.91804
7	C	4.074877	-2.77308	0.45967
8	C	3.155107	-3.77292	0.867556
9	C	1.930989	-3.44797	1.385318
10	C	1.509375	-2.10252	1.492505
11	C	0.287583	-1.71274	2.148823
12	C	5.389705	-3.09655	-0.04981
13	C	-0.4011	-2.58409	3.025614
14	C	-1.50366	-2.16488	3.733836
15	C	-1.94917	-0.83798	3.599773
16	C	-1.31919	0.017892	2.726001

17	C	-0.2033	-0.39219	1.949593
18	C	6.291817	-2.04142	-0.39159
19	C	7.58088	-2.37537	-0.88916
20	C	7.970023	-3.68385	-1.04782
21	C	7.081496	-4.72359	-0.71513
22	C	5.826466	-4.43148	-0.23
23	C	5.894194	-0.67664	-0.22976
24	C	0.49214	0.546679	1.06195
25	C	-5.6895	-0.19477	-0.56296
26	C	-0.37221	1.584535	0.4331
27	C	-0.89224	5.315764	0.087235
28	C	0.357826	5.768438	-0.27122
29	C	1.364578	4.832673	-0.54479
30	C	1.13366	3.488649	-0.3386
31	C	-0.09773	2.99584	0.155138
32	C	-6.79832	0.694085	-0.3816
33	C	-8.13498	0.240865	-0.5478
34	C	-9.20549	1.084385	-0.3669
35	C	-8.9863	2.427436	-0.00927
36	C	-7.70218	2.896128	0.158598
37	C	-1.17095	3.93875	0.248887
38	C	-6.57504	2.058727	-0.01989
39	C	-5.21913	2.536341	0.14431
40	C	-4.92378	3.876272	0.502505
41	C	-3.63442	4.327803	0.601373
42	C	-2.53474	3.471089	0.364981
43	C	-4.39006	0.265487	-0.38239
44	C	-4.13597	1.64307	-0.04198
45	C	-2.78759	2.106071	0.108984
46	C	-1.68826	1.172616	0.101465
47	C	-1.99134	-0.18685	-0.26757
48	C	-3.25061	-0.603	-0.53439
49	C	6.861843	0.423168	-0.55212
50	C	-5.94252	-1.62449	-0.94056
51	C	7.758671	0.884324	0.431325
52	C	8.654537	1.9063	0.109116
53	C	8.689061	2.484766	-1.15983
54	C	7.795893	2.009855	-2.12027
55	C	6.881831	0.991429	-1.84047
56	C	-6.10336	-2.60215	0.06011
57	C	-6.34768	-3.92521	-0.31505
58	C	-6.43464	-4.30915	-1.65321
59	C	-6.27448	-3.32475	-2.6285
60	C	-6.02975	-1.99	-2.29791
61	C	7.762319	0.293502	1.822318
62	C	5.936889	0.513865	-2.91885
63	C	9.645952	3.609318	-1.47596
64	C	-5.86007	-0.96377	-3.3942
65	C	-6.0134	-2.24092	1.524676
66	C	-6.66717	-5.7515	-2.03516
67	H	2.660955	2.307033	0.984902
68	H	4.926024	1.770199	0.381847
69	H	3.4223	-4.81789	0.79241
70	H	1.266874	-4.24213	1.70133
71	H	-0.02229	-3.58577	3.183224
72	H	-2.0019	-2.84232	4.417888
73	H	-2.781	-0.47994	4.195587
74	H	-1.66652	1.038283	2.648074

75	H	8.260004	-1.57282	-1.14754
76	H	8.95757	-3.91507	-1.4308
77	H	7.383671	-5.75699	-0.84216
78	H	5.16406	-5.25168	0.011455
79	H	-1.6978	6.031893	0.180636
80	H	0.537013	6.828528	-0.40849
81	H	2.317217	5.156257	-0.94915
82	H	1.890112	2.788489	-0.64587
83	H	-8.30095	-0.79333	-0.82127
84	H	-10.2164	0.715597	-0.49923
85	H	-9.82793	3.095791	0.133366
86	H	-7.56399	3.93398	0.430398
87	H	-5.72765	4.573903	0.693539
88	H	-3.4651	5.362962	0.867216
89	H	-1.1704	-0.87988	-0.38854
90	H	-3.42029	-1.62014	-0.86353
91	H	9.344462	2.256615	0.871356
92	H	7.810092	2.440354	-3.11737
93	H	-6.4747	-4.67401	0.461448
94	H	-6.34302	-3.59985	-3.67702
95	H	7.96779	-0.78051	1.801626
96	H	8.521884	0.770899	2.444021
97	H	6.793734	0.421034	2.314311
98	H	6.130157	1.031397	-3.86025
99	H	6.039271	-0.56053	-3.09521
100	H	4.893276	0.691749	-2.64371
101	H	9.196888	4.582878	-1.25
102	H	10.5634	3.529592	-0.88808
103	H	9.919963	3.614401	-2.53353
104	H	-5.98492	-1.42231	-4.37685
105	H	-6.58932	-0.1539	-3.30286
106	H	-4.86863	-0.50288	-3.35991
107	H	-5.0191	-1.86418	1.781884
108	H	-6.72977	-1.45833	1.789826
109	H	-6.21607	-3.11181	2.150898
110	H	-7.24695	-6.27751	-1.27302
111	H	-7.20264	-5.83157	-2.98414
112	H	-5.71744	-6.28572	-2.14995

5. Optical resolution by HPLC

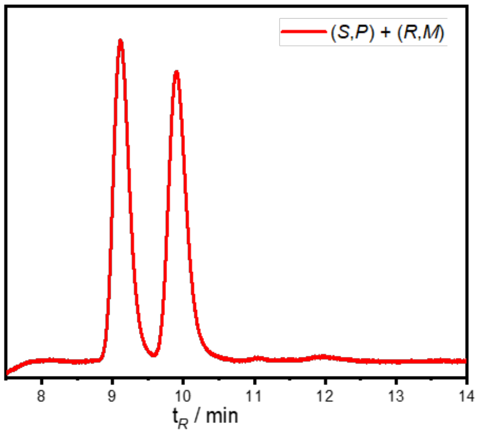

Figure S3. (a) Chiral HPLC traces during the separation of **6** monitored at 360 nm. A mixture of n-hexane/toluene (13:7) was used as the eluent with a flow rate of 0.5 mL/min by Daicel Chiralpak IG HPLC column.

6. Absorption spectra

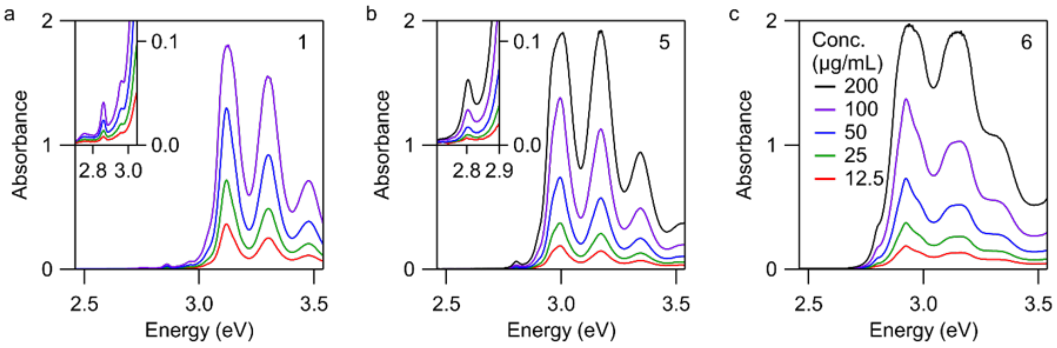

Figure S4. Concentration-dependent absorption spectra for (a) **1**, (b) **5**, and (c) **6** in toluene. These spectra were used to calculate the molar absorptivity (main-text Figure 3a). The peaks assigned to S_1 vary in precise ratio with the stronger S_2 peaks, indicating that they do not arise from aggregates formed at high concentration.

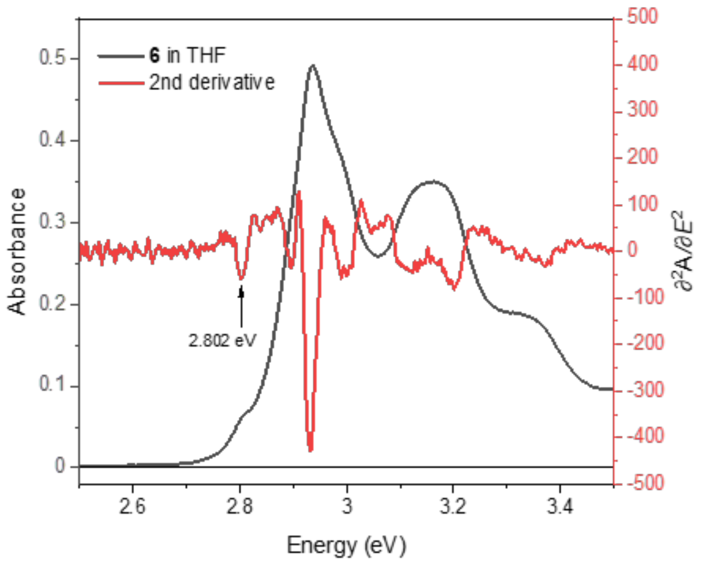

Figure S5. Steady-state absorption spectrum **6** in THF and its second derivative. The black arrow indicates the S_1 energy level of which is used to calculate the driving force of charge transfer.

7. NMR and MS spectra

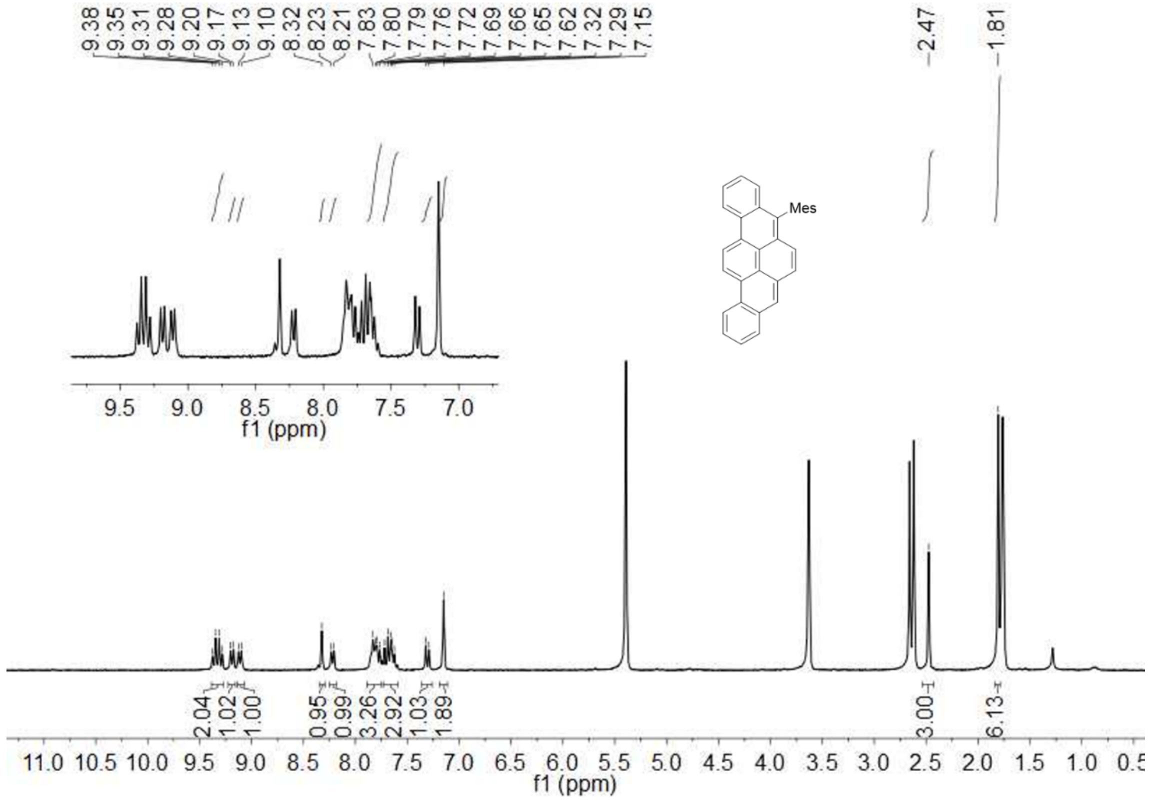

Figure S6 ^{1}H NMR spectrum of compound **3** in THF-d_8 (300 MHz, 298 K).

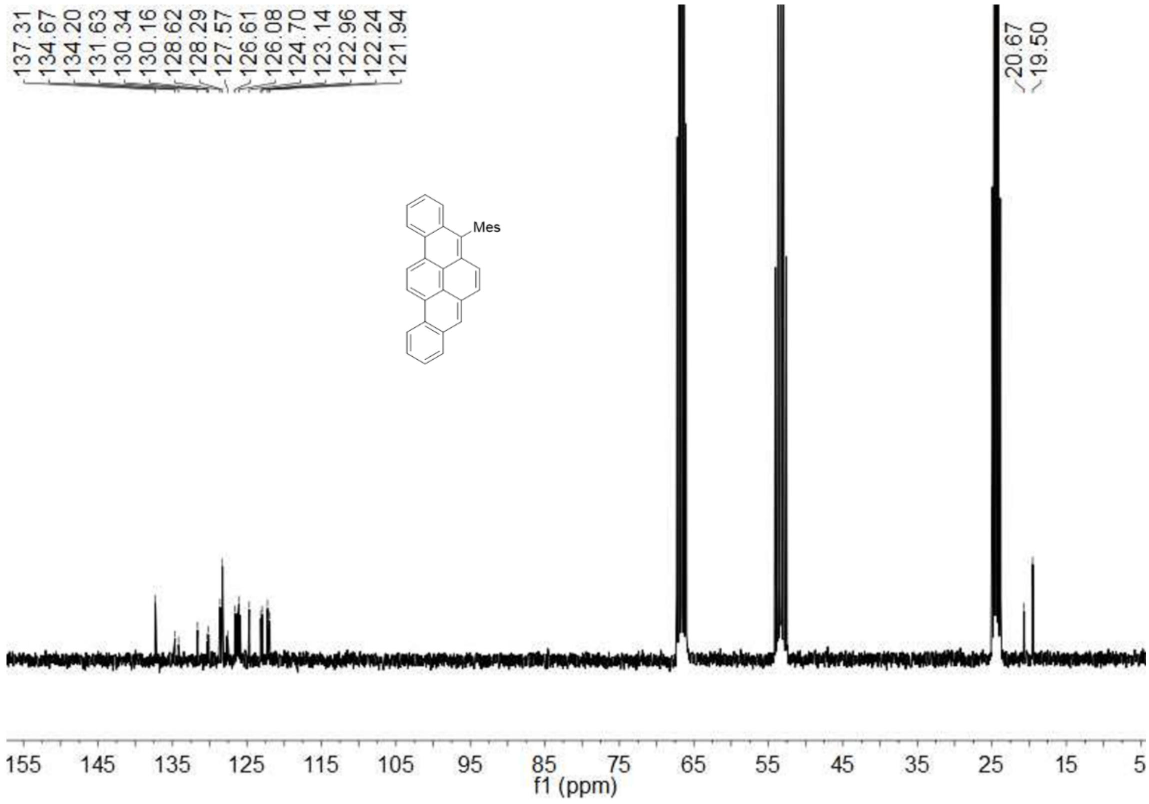

Figure S7 ^{13}C NMR spectrum of compound **3** in THF-d_8 (75 MHz, 298 K).

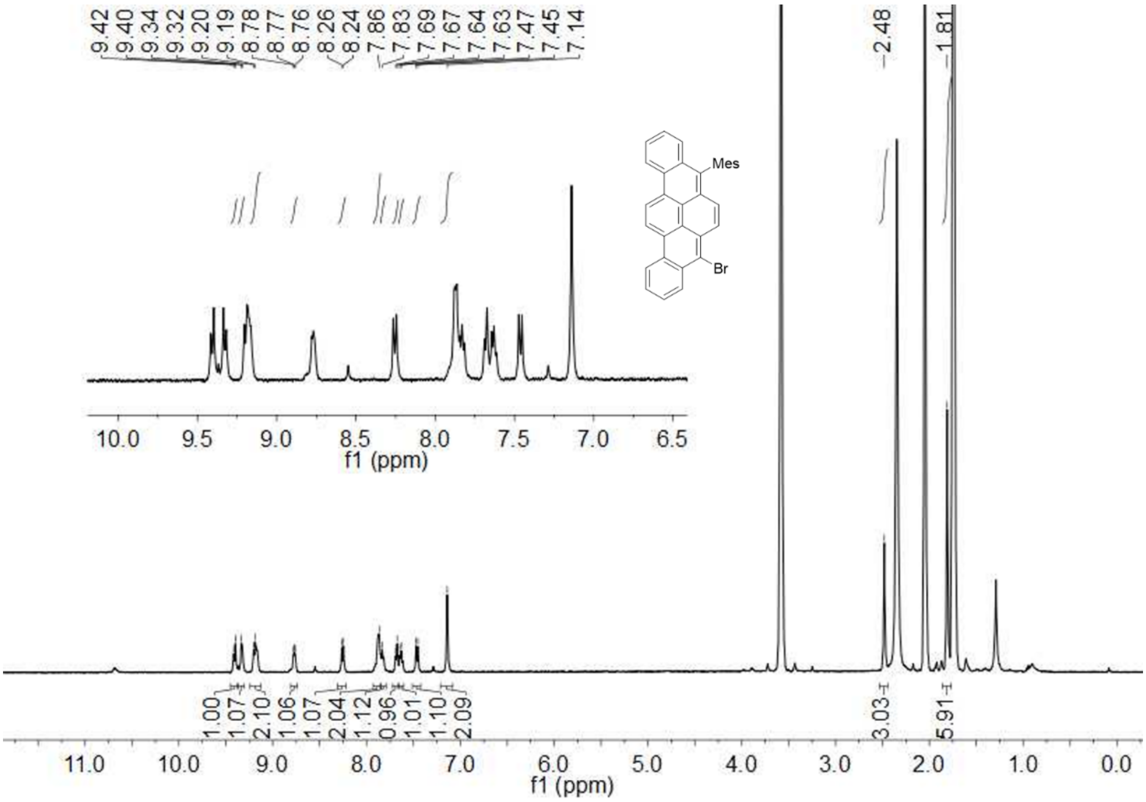

Figure S8 ^{1}H NMR spectrum of compound **4** in THF-d_8 (500 MHz, 298 K).

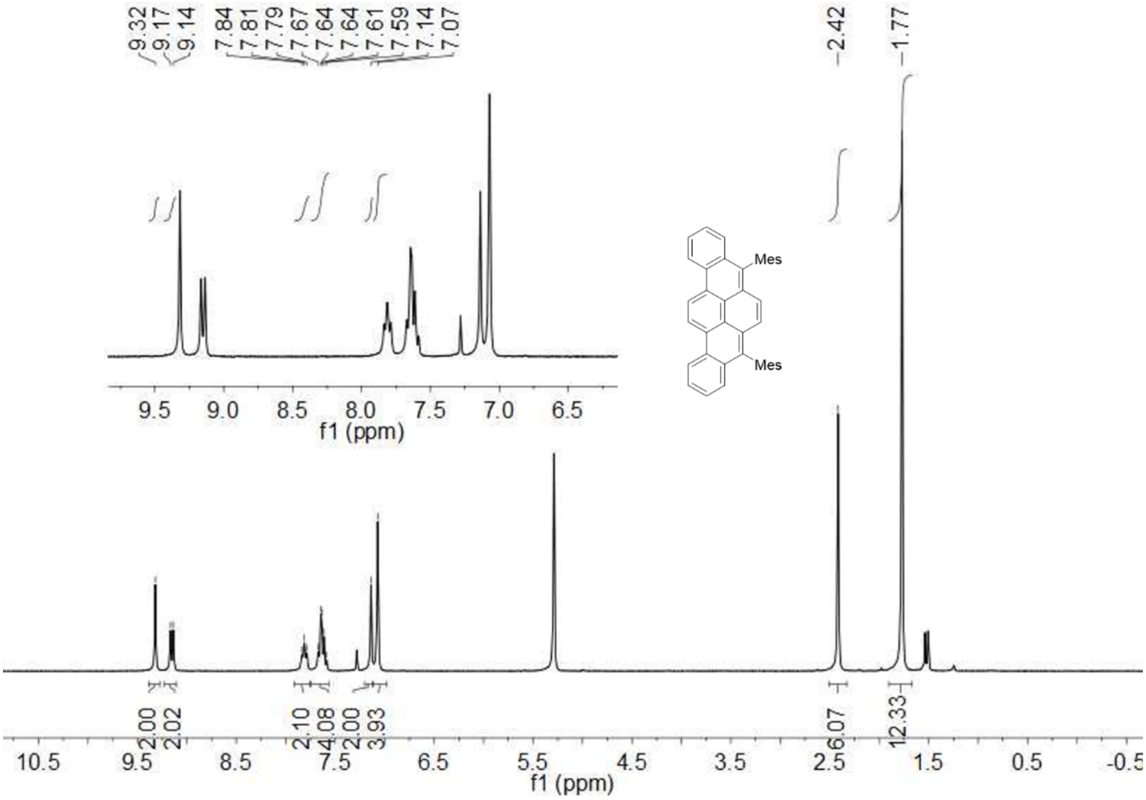

Figure S9 ^{1}H NMR spectrum of compound **5** in $CDCl_3$ (300 MHz, 298 K).

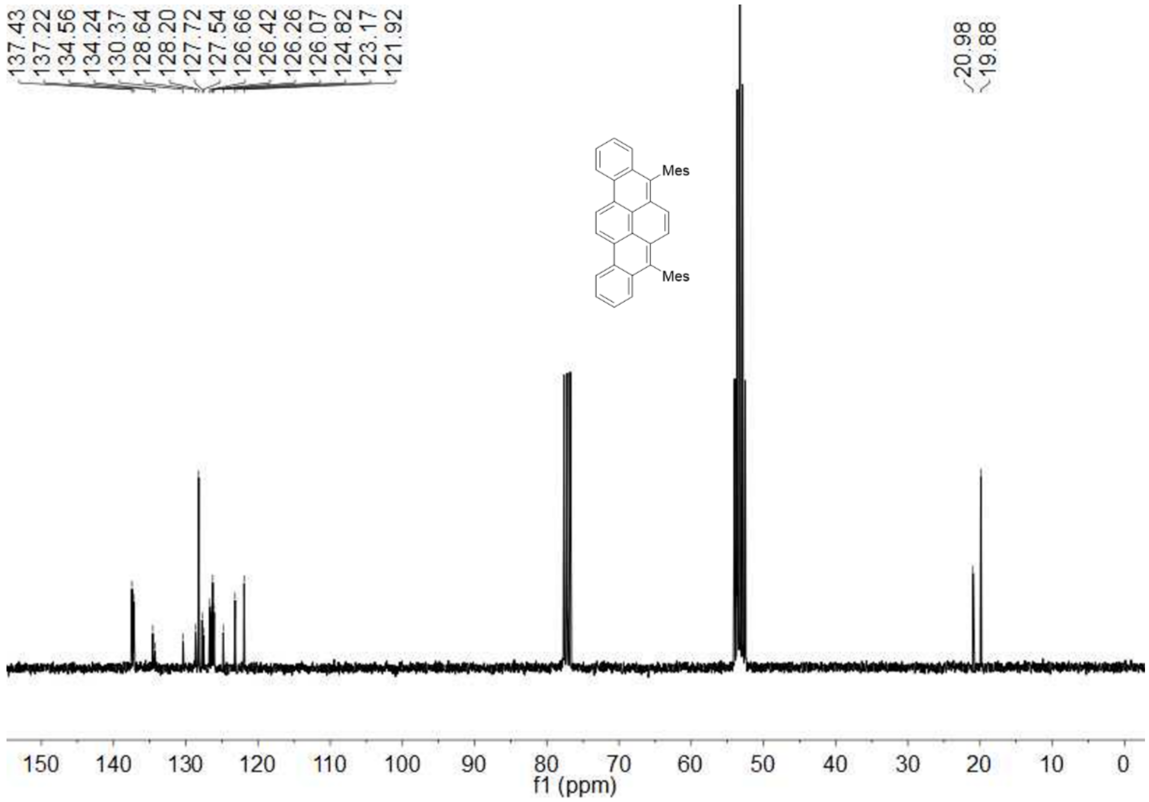

Figure S10 ^{13}C NMR spectrum of compound **5** in $CDCl_3$ (75 MHz, 298 K).

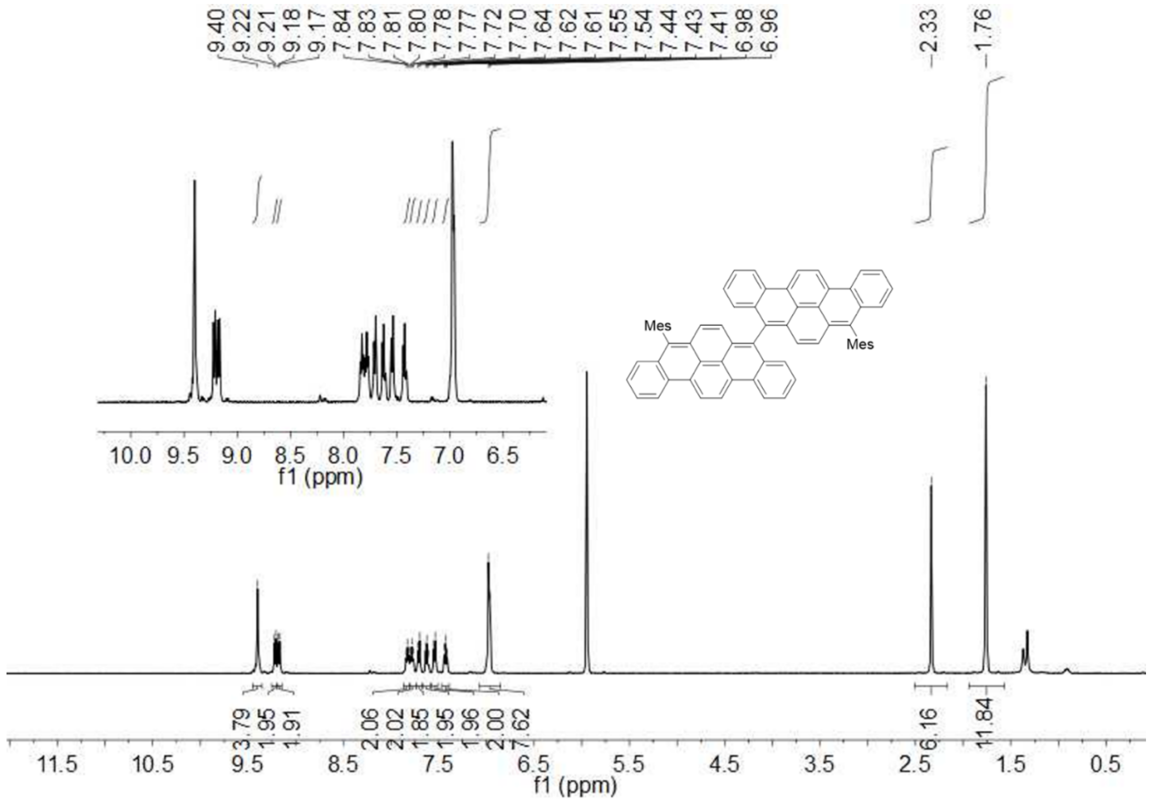

Figure S11 ^{1}H NMR spectrum of compound **6** in $C_2D_2Cl_4$ (500 MHz, 403 K).

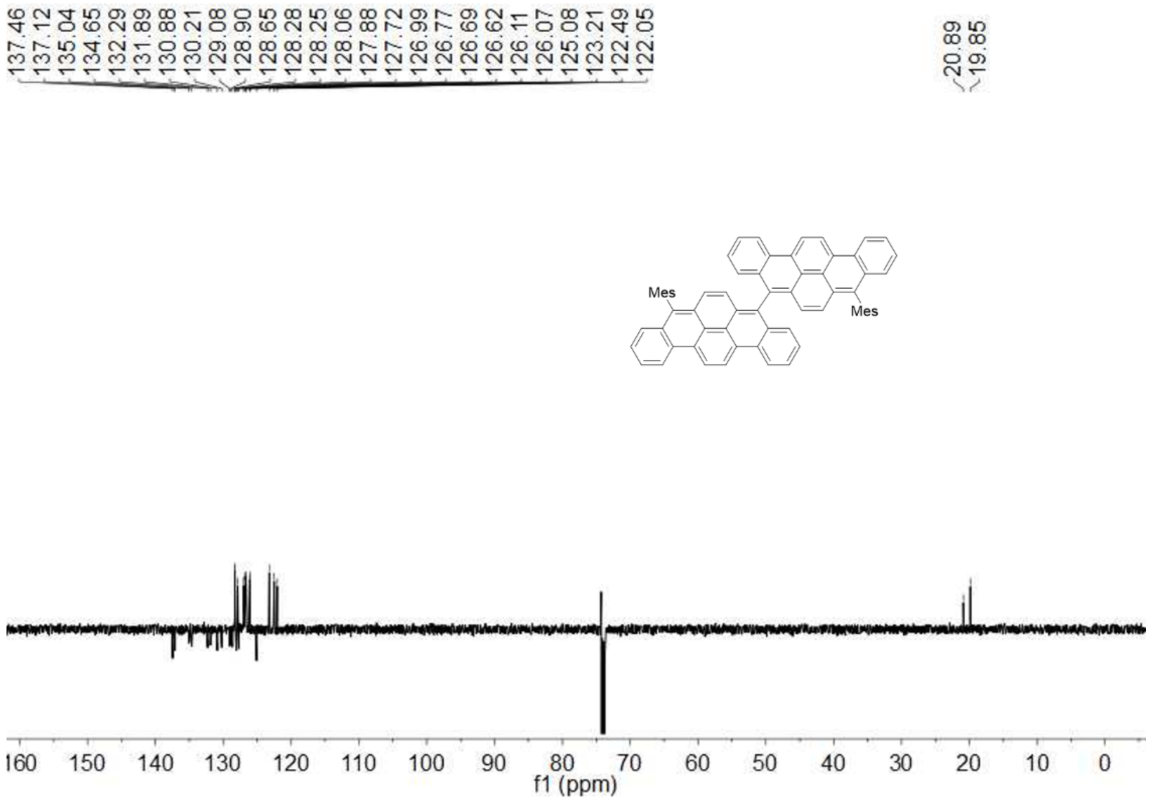

Figure S12 ^{13}C NMR spectrum of compound **6** in $C_2D_2Cl_4$ (126 MHz, 403 K).

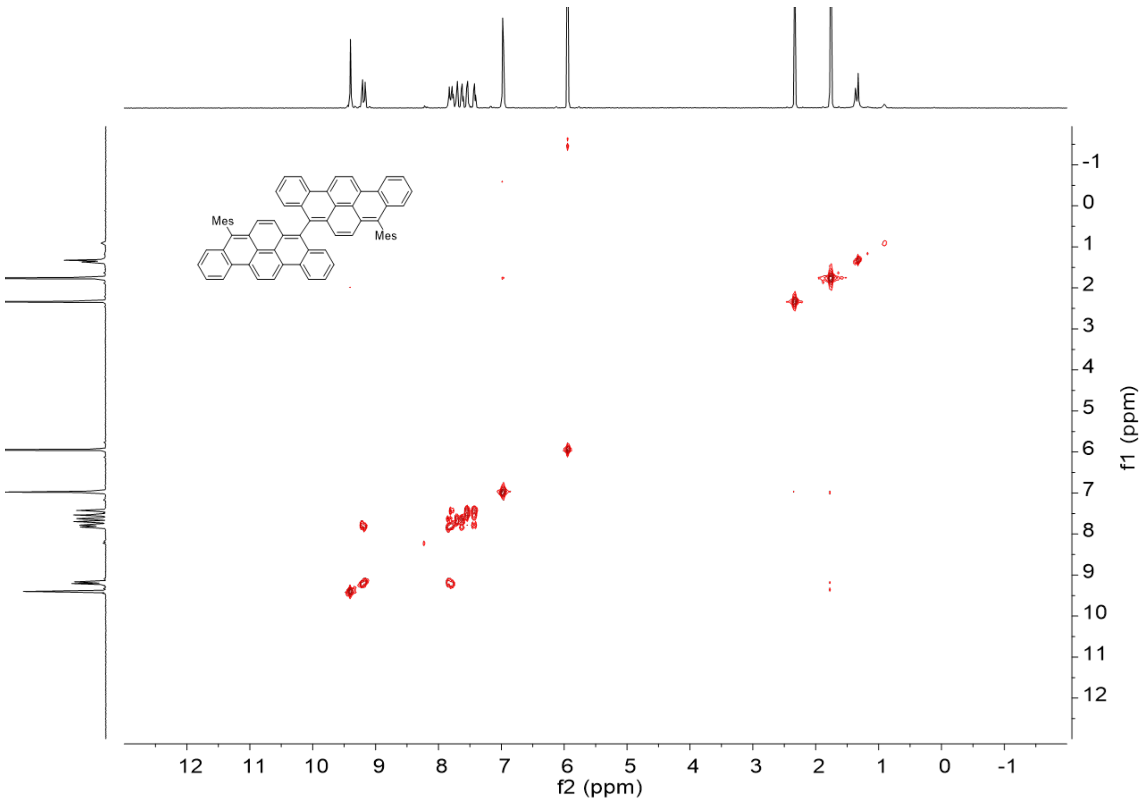

Figure S13 ^{1}H,^{1}H-COSY NMR spectrum of compound **6** in $C_2D_2Cl_4$ (500 MHz, 403 K).

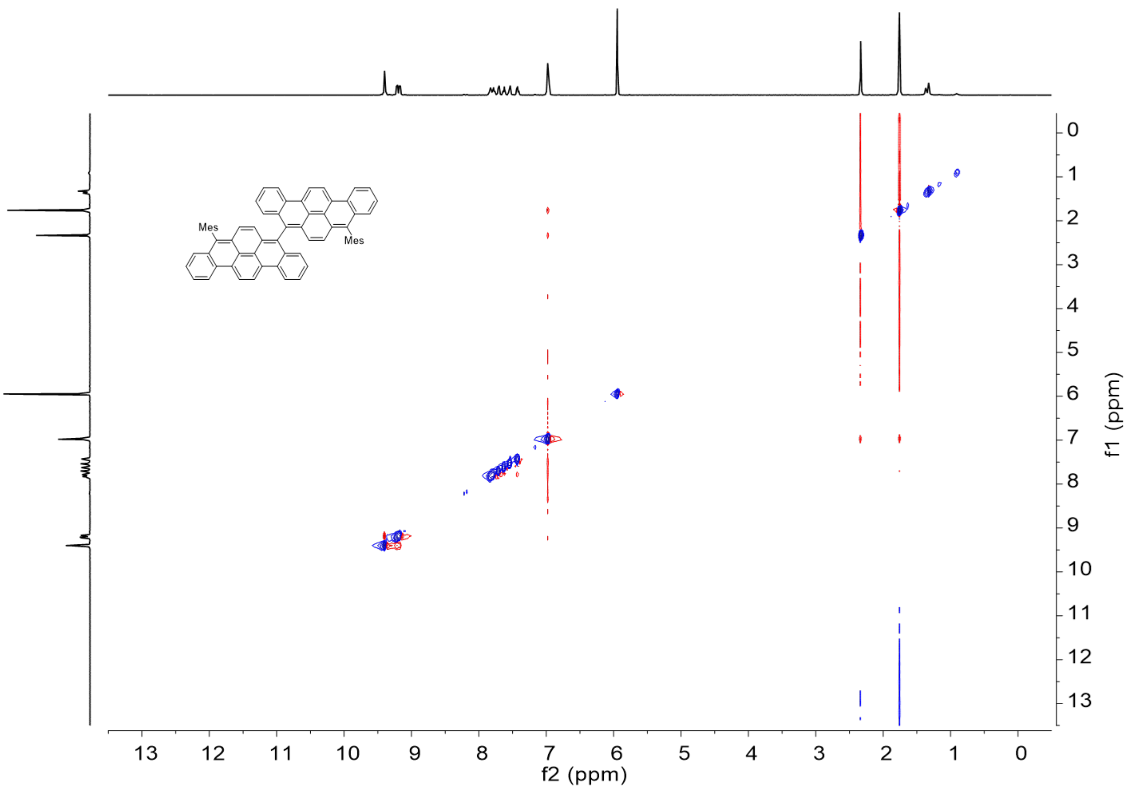

Figure S14 ^{1}H,^{1}H-NOESY NMR spectrum of compound **6** in $C_2D_2Cl_4$ (500 MHz, 403 K).

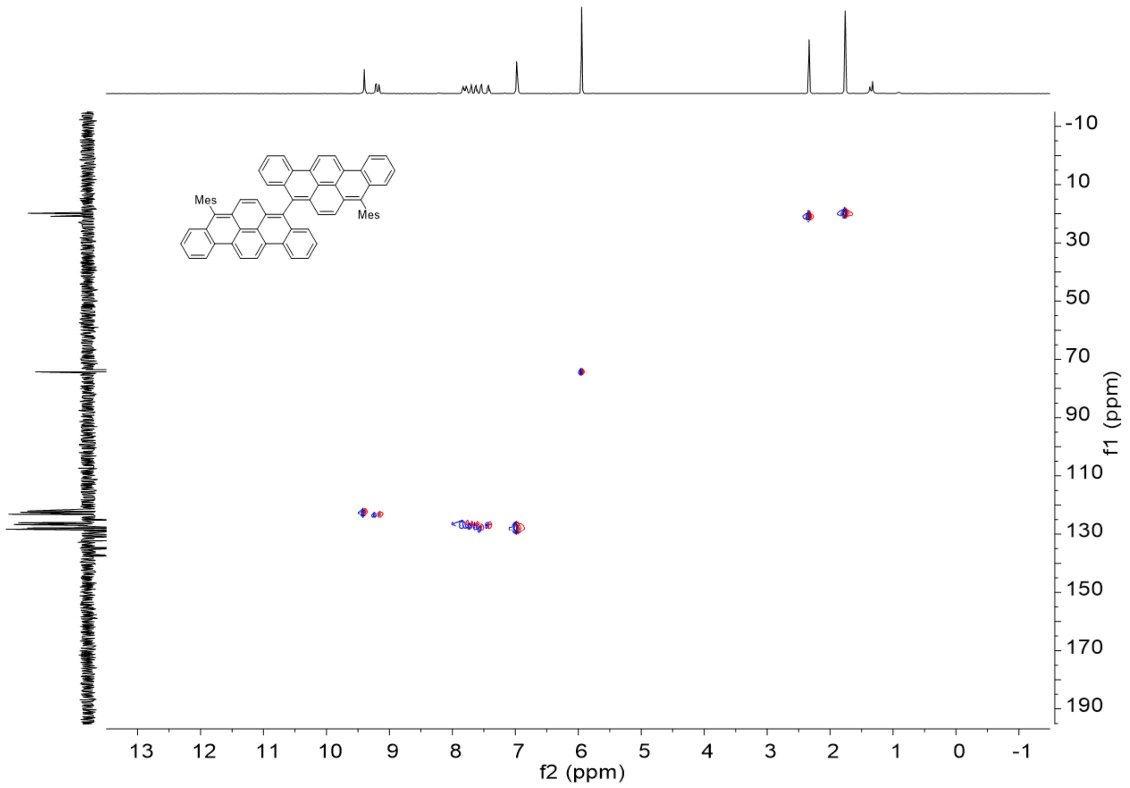

Figure S15. ^{1}H-^{13}C COSY NMR spectrum of compound **6** in $C_2D_2Cl_4$ (126 MHz, 403 K).

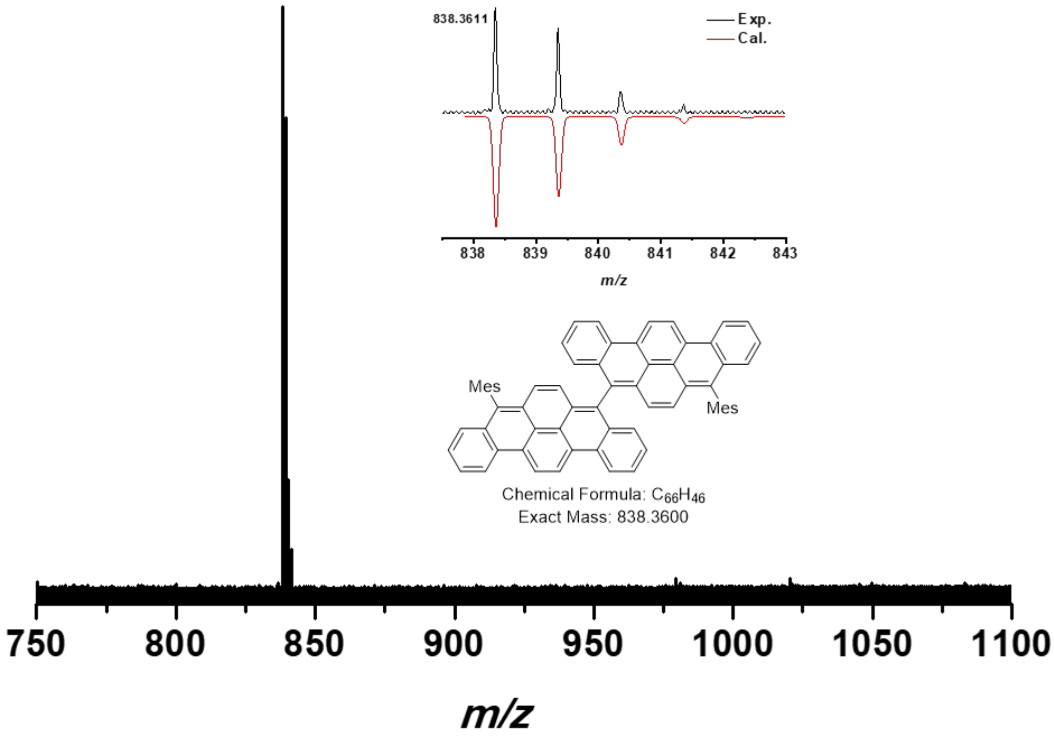

Figure S16. HR MALDI-TOF mass spectrum and isotopic distribution pattern of compound **6**.

Reference

[S1] J. C. deMello, H. F. Wittmann, R. H. Friend, *Adv. Mater.* **1997**, *9*, 230.

[S2] X. S. Xu, A. Kinikar, M. Di Giovannantonio, P. Ruffieux, K. Müllen, R. Fasel, A. Narita, *Bull. Chem. Soc. Jpn.* **2021**, *94*, 997.

[S3] R. D. Gaussian 09, Frisch, M. J.; Trucks, G. W.; Schlegel, H. B.; Scuseria, G. E.;; Robb, M. A. C., J. R.; Scalmani, G.; Barone, V.; Mennucci, B.; Petersson, G. A.;; Nakatsuji, H. C., M.; Li, X.; Hratchian, H. P.; Izmaylov, A. F.; Bloino, J.; Zheng, G.;; Sonnenberg, J. L. H., M.; Ehara, M.; Toyota, K.; Fukuda, R.; Hasegawa, J.; Ishida, M.;; Nakajima, T. H., Y.; Kitao, O.; Nakai, H.; Vreven, T.; Montgomery, Jr., J. A.; Peralta, J.; E.; Ogliaro, F. B., M.; Heyd, J. J.; Brothers, E.; Kudin, K. N.; Staroverov, V. N.;; Kobayashi, R. N., J.; Raghavachari, K.; Rendell, A.; Burant, J. C.; Iyengar, S. S.;; Tomasi, J. C., M.;Rega, N.; Millam, N. J.; Klene, M.; Knox, J. E.; Cross, J. B.; Bakken,; V.; Adamo, C. J., J.; Gomperts, R.; Stratmann, R. E.; Yazyev, O.; Austin, A. J.;; Cammi, R. P., C.; Ochterski, J. W.; Martin, R. L.; Morokuma, K.; Zakrzewski, V. G.;; Voth, G. A. S., P.; Dannenberg, J. J.; Dapprich, S.; Daniels, A. D.; Farkas, Ö.;Foresman, J. B.; Ortiz, J. V.; Cioslowski, J.; Fox, D. J. Gaussian, Inc., Wallingford CT, 2013.
